# From Traditional Remedy to Evidence-Based Phytotherapeutic Agent: *Hedera helix* L. in Respiratory Medicine

**DOI:** 10.3390/plants15172610

**Published:** 2026-08-27

**Authors:** Agata Maria Pawłowska

**Affiliations:** Institute of Food Technology and Nutrition, Faculty of Technology and Life Sciences, University of Rzeszow, 4 Zelwerowicza St., 35-601 Rzeszow, Poland; agpawlowska@ur.edu.pl

**Keywords:** *Hedera helix*, ivy leaf, productive cough, acute bronchitis, α-hederin, hederacoside C, pharmacology, toxicology, clinical efficacy, safety

## Abstract

*Hedera helix* L. leaf extracts are regulated expectorants for productive cough, yet their evidence is dispersed across pharmaceutical quality, nonclinical pharmacology, disposition, toxicology, clinical pharmacology, efficacy, safety, and regulatory assessment. This review integrates those domains in a single study-level account and explicitly distinguishes isolated constituents, incompletely specified extracts, defined monograph preparations, proprietary extracts, and combination products. Hederacoside C is the pharmacopoeial marker, while α-hederin is the constituent most extensively examined in β2-adrenergic receptor models. Cell studies documented reduced agonist-induced receptor internalisation, greater ligand binding and cAMP responsiveness, and altered GRK2/β-arrestin signalling. Isolated tissue and animal studies reported antispasmodic, anti-inflammatory, antitussive, and tracheobronchial secretory effects. Rat studies showed low, matrix-dependent oral exposure to hederacoside C and α-hederin. Small exploratory human studies detected no or only trace α-hederin and did not permit conventional pharmacokinetic analysis. Clinical evidence includes placebo-controlled adult trials, active comparator studies, paediatric investigations, postmarketing cohorts, systematic reviews, and pharmacovigilance reports. Controlled adult trials of EA 575 reported greater short-term improvement in cough or Bronchitis Severity Score than placebo; paediatric efficacy evidence is dominated by observational studies and small airway function trials. Short-term tolerability was generally favourable, with gastrointestinal and hypersensitivity reactions as the principal recognised adverse effects. The European Union monograph recognises specified extracts for productive cough, contraindicates use below two years, and does not recommend use during pregnancy or lactation. The distinctive contribution of this review is the complete quality-to-clinic evidence map, including dose, model, endpoint, study design, extract identity, and unresolved evidence domain for each major dataset.

## 1. Introduction

Ivy leaf preparations are established herbal medicines in European respiratory care. The official drug is the dried leaf of *Hedera helix* L., and the European Union herbal monograph lists defined oral extracts for expectorant use in productive cough. Syrups, drops, tablets, and other dosage forms are marketed for adults and children within product-specific age and dose limits. The monograph specifies preparation categories through the drug–extract ratio and extraction solvent [1,2,3].

Research on *H. helix* covers several evidence domains. Pharmacognostic studies have identified triterpenoid saponins, flavonoids, phenolic acids, coumarins, polyacetylenes, sterols, and minor volatile constituents. Experimental pharmacology has focused on α-hederin and β2-adrenergic receptor regulation, inflammatory pathways, airway secretion, cough models, and a wider group of antimicrobial, antiviral, antifungal, antiparasitic, antioxidant, and hepatic effects. Animal studies have examined the disposition of hederacoside C, hederacoside D, and α-hederin [4,5,6,7,8,9].

The clinical literature includes placebo-controlled trials, randomised comparisons between herbal preparations, observational studies, postmarketing cohorts, paediatric investigations, systematic reviews, and an individual participant meta-analysis. Adult trials have mainly studied acute cough or acute bronchitis. Paediatric reports include acute bronchitis, productive cough, and add-on treatment in bronchial asthma. Clinical safety information is derived from these studies, spontaneous reports, case reports, and regulatory review [10,11,12,13,14,15,16,17,18].

The distinction from previous comprehensive reviews warrants explicit statement. Existing reviews have provided broad compilations of the botany, phytoconstituents, and pharmacological activities of *Hedera* species; product-specific summaries of the clinical evidence for a single proprietary extract; and systematic appraisals of controlled clinical trials [16,19,20,21,22,23,24,25,26]. The present article differs from these publications in scope and method. It is restricted to *H. helix* leaf as a medicinal material defined within the European regulatory and pharmacopoeial framework and consistently distinguishes evidence obtained with isolated constituents, chemically defined extracts, incompletely specified preparations, and combination products.

The review integrates pharmaceutical quality, nonclinical pharmacology, pharmacokinetics, toxicology, adult and paediatric clinical efficacy, clinical safety, and regulatory assessment at the level of individual studies. Study-level pharmacokinetic and toxicological tables are accompanied by clinical tables stratified according to study design, allowing randomised controlled trials, observational studies, and postmarketing evidence to be evaluated separately. The evidence base is updated through June 2026 and includes the recent preclinical antitussive and mucoactive program, the three-arm comparative clinical trial, the paediatric prescription database analysis, and the 2025 EMA periodic review call. Each major evidence domain concludes with an explicit synthesis of what can presently be considered established, what remains unresolved, and which limitations impede translation into clinical practice. The novelty of the article therefore lies in consolidating the complete quality-to-clinic evidence chain at the study level rather than providing another catalogue of constituents or pharmacological activities (Table 1).

## 2. Literature Search Strategy

This article is a structured but non-systematic (narrative) review. The literature was identified through a comprehensive, reproducible search, but a formal systematic review process—including dual independent screening and complete record-level accounting—was not undertaken; sources were selected by the author on the basis of relevance to the scope of the review.

PubMed/MEDLINE and Europe PMC were searched from database inception to 30 June 2026, complemented by hand searching of regulatory and pharmacopoeial sources (the EMA/HMPC herbal medicines database and the European Pharmacopoeia) and of the reference lists of retrieved articles and previous reviews. The search combined, in title/abstract fields and adapted to each database, the terms “Hedera helix”, “ivy leaf”, “Hederae helicis folium”, hederacoside C, α-hederin, hederagenin, and “EA 575” with terms covering phytochemistry, extraction, β2-adrenergic receptor activity, pharmacodynamics, pharmacokinetics, metabolism, toxicology, genotoxicity, reproductive toxicity, cough, bronchitis, asthma, clinical trials, efficacy, safety, and adverse events.

The search retrieved 1033 records in PubMed/MEDLINE and 1154 in Europe PMC (2187 in total; 1255 after removal of duplicates in a reference manager). From this pool, together with the regulatory, pharmacopoeial, and reference list sources, the author selected studies relevant to the medicinal use of *H. helix* leaf and its defined constituents and extracts, spanning phytochemistry, pharmacology, pharmacokinetics, toxicology, clinical efficacy, safety, and regulation; a total of 97 sources were ultimately cited. Records concerning unrelated topics—for example, ecological, agronomic, horticultural, animal-feed, bioremediation, plant molecular biology, or other-species studies without relevance to the medicinal use of ivy leaf—were not considered further. Consistent with a narrative review, studies are summarised at the level of individual reports, and interpretation is concentrated in the knowledge gaps section; no quantitative meta-analysis or formal risk-of-bias synthesis was performed.

## 3. Botanical Characteristics, Taxonomy, and Distribution

*Hedera helix* L. belongs to the family Araliaceae and the order Apiales (Table 2). Hedera is a genus of evergreen climbers distributed mainly in Europe, North Africa, and Western Asia. The accepted medicinal species is *Hedera helix* L.; related taxa and horticultural forms require distinction during raw material authentication. Current taxonomic status and geographic distribution are maintained in Plants of the World Online [19,26,27].

*Hedera helix* is an evergreen woody climber that attaches to trees, rocks, and built surfaces through adventitious rootlets. Juvenile vegetative shoots carry alternate leathery leaves with three to five lobes, whereas adult flowering shoots carry unlobed ovate or rhomboid leaves. Small greenish-yellow flowers occur in umbels, and dark berries mature after flowering. The heterophylly of juvenile and adult shoots is a characteristic feature used in botanical description [19,26].

The pharmacopoeial drug is prepared from dried leaves rather than berries or unspecified aerial parts. Botanical authentication includes macroscopic and microscopic characteristics, chromatographic identity, and purity testing. Studies of fresh leaves or berries, and case reports involving accidental plant ingestion, represent exposures different from medicinal leaf extracts [2,3].

The native range extends through most of Europe and parts of the Mediterranean region and Western Asia; the species has been introduced widely elsewhere. An investigation of ivy leaves from different geographic origins measured phenolic acids, triterpenoid saponins, amino acids, and antioxidant activity and identified phytogeographic variation. Analytical comparisons of leaves, dry extract, and pharmaceutical products have also documented differences and similarities in component profiles [27,28,29].

Season, developmental stage, leaf type, drying conditions, storage, extraction solvent, and manufacturing process influence the measured constituent profile. These variables are controlled through qualified starting material, defined extraction, marker assays, chromatographic fingerprints, and finished product specifications [4,30,31].

Evidence synthesis. Taken together, pharmacopoeial and botanical sources establish the medicinal material as the whole or cut dried leaf of *H. helix*, distinct from berries, fresh leaf exposures, and related taxa. Geographic origin, season, developmental stage, and post-harvest processing modify measured constituent profiles, although their quantitative effects on finished extract performance remain incompletely defined. Transfer of botanical data to medicinal products therefore requires authenticated leaf material together with a specified extraction and manufacturing process [2,3,4,27,28,29,30,31].

## 4. Phytochemistry and Chemical Constituents

**Figure 1:**
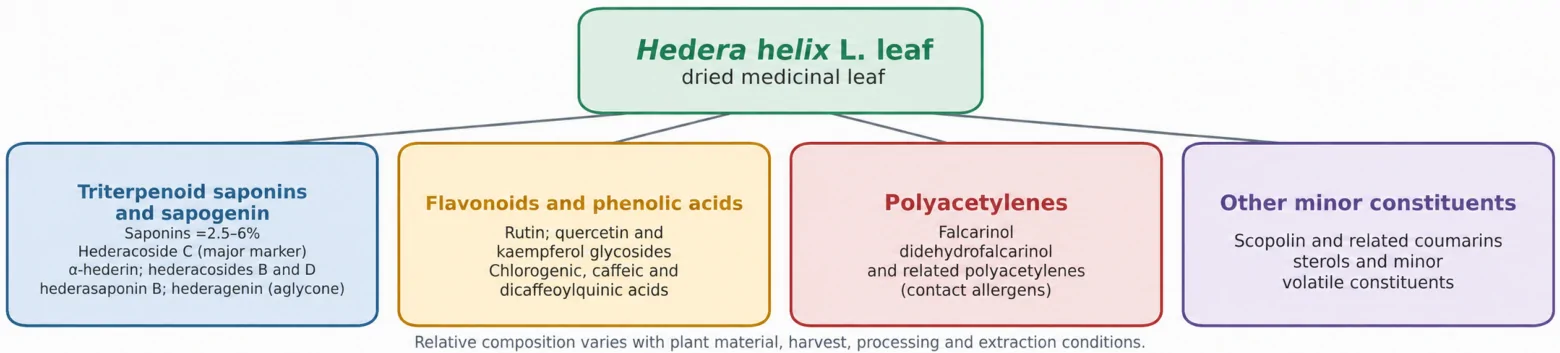
Principal phytochemical groups of *Hedera helix* dried medicinal leaf. Triterpenoid saponins (≈2.5–6% of the leaf) are the dominant, pharmacologically defining class, comprising the bisdesmosidic pharmacopoeial marker hederacoside C (minimum 3.0% in the dried drug), hederacosides B and D, hederasaponin B, the monodesmosidic α-hederin—formed from hederacoside C by ester hydrolysis and the constituent most extensively studied in β_2_-adrenergic receptor and membrane models—and the corresponding oleanane-type aglycone hederagenin. The polyacetylenes falcarinol and didehydrofalcarinol are the principal contact allergens of the fresh plant. Relative composition varies with plant material, harvest, processing, and extraction conditions.

Analytical investigations have identified triterpenoid saponins as the dominant specialised metabolite group in ivy leaf. Hederacoside C is usually the major saponin and the pharmacopoeial marker. Other identified saponins include hederacosides B and D, hederasaponin B, α-hederin, and related hederagenin glycosides. Non-saponin constituents include flavonoids, chlorogenic and other phenolic acids, scopolin and related coumarins, polyacetylenes, sterols, and minor volatile compounds (Figure 1, Table 3) [4,5,19,25].

**Figure 2:**
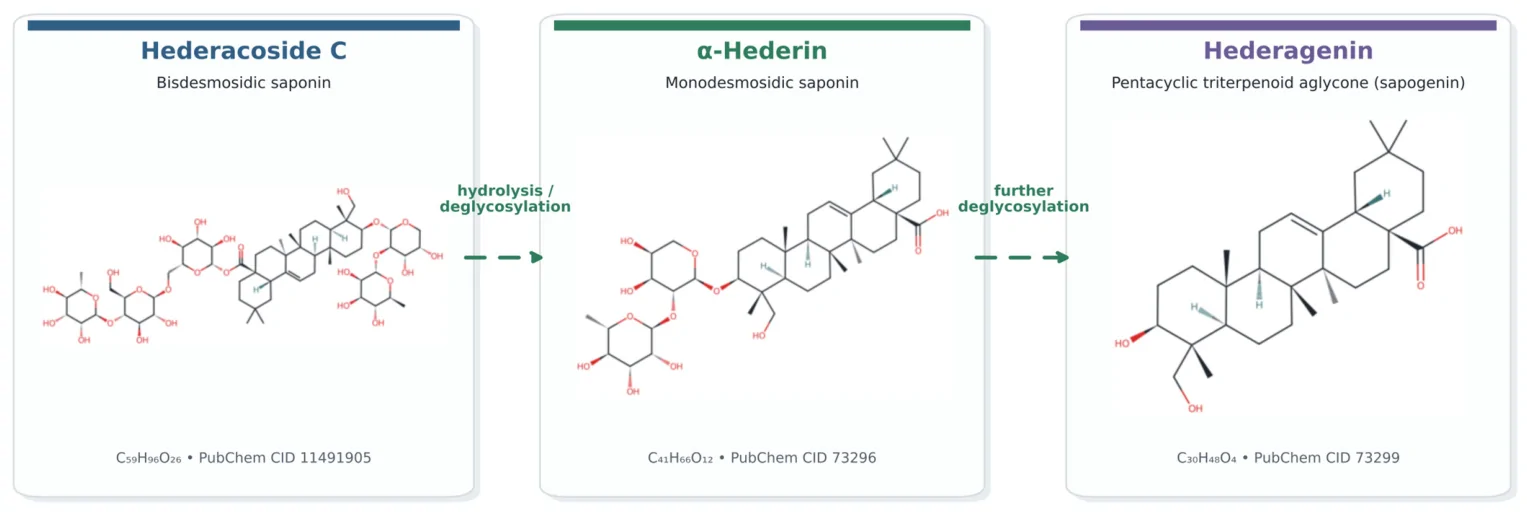
Structural and hydrolytic relationships among the principal triterpenoids of *Hedera helix* L. leaf. Hydrolysis and deglycosylation relate the bisdesmosidic saponin hederacoside C to the monodesmosidic saponin α-hederin; further deglycosylation yields hederagenin, the corresponding pentacyclic triterpenoid aglycone, or sapogenin. The dashed arrows indicate structural and hydrolytic relationships and should not be interpreted as evidence of a quantitatively established metabolic sequence in humans. Structures were generated from PubChem 2D records: hederacoside C (CID 11491905), α-hederin (CID 73296), and hederagenin (CID 73299).

Hederacoside C is a bisdesmosidic oleanane-type saponin derived from hederagenin. It occurs at higher concentrations than α-hederin in most raw leaf and extract profiles and is quantified for release testing. α-Hederin is a monodesmosidic derivative that can be present in the extract or formed through deglycosylation of hederacoside C. Hederagenin is the corresponding aglycone (Figure 2). The three compounds have been compared directly in β2-adrenergic receptor experiments [4,6,30].

HPLC-DAD and LC–MS/MS methods have been reported for simultaneous measurement of saponins and flavonoids in ivy extracts and pharmaceutical products. The analytical studies measured batch-to-batch and product-to-product profiles and supplied methods for hederacoside C and α-hederin quantification [5,29,30].

Reported flavonoids include rutin and glycosides of quercetin and kaempferol. Phenolic constituents include chlorogenic, caffeic, and dicaffeoylquinic acids. A phenolic fraction was used in a mouse model of lipopolysaccharide-induced acute lung injury, where inflammatory and oxidative endpoints were measured. These studies identify non-saponin material with experimental activity [5,28,32].

Scopolin and related coumarins occur in small amounts. Falcarinol, didehydrofalcarinol, and related polyacetylenes have been identified in fresh ivy and investigated as contact allergens. Sterols and volatile constituents have also been reported, although they are minor components of medicinal leaf preparations [19,26,33,34].

**Table 3:** Principal phytochemical groups identified in *Hedera helix* leaf.

Group	Representative Compounds	Analytical or Experimental Relevance	Reference
Bisdesmosidic saponins	Hederacoside C; hederacosides B and D	Major leaf constituents; hederacoside C is the pharmacopoeial marker	[3,4]
Monodesmosidic saponins	α-Hederin and related hederins	Measured in extracts; studied in β2 receptor and toxicological models	[6,30,35]
Sapogenins	Hederagenin; oleanolic acid	Aglycones and potential metabolites	[4,9]
Flavonoids	Rutin; quercetin and kaempferol glycosides	Chromatographic fingerprint and antioxidant assays	[5,28]
Phenolic acids	Chlorogenic, caffeic, dicaffeoylquinic acids	Fingerprinting; phenolic fraction lung injury study	[28,32]
Polyacetylenes	Falcarinol; didehydrofalcarinol	Fresh plant contact allergy	[33,34]
Other minor groups	Scopolin, sterols, volatile constituents	Completeness of phytochemical profile	[19,26]

The EU monograph lists three dry extracts, one liquid extract, and one soft extract with defined drug–extract ratios and extraction solvents (Table 4). Laboratory and green extraction studies show that ethanol proportion, temperature, duration, particle size, and extraction technique alter yield and constituent recovery. Pharmaceutical products also differ in dosage form, extract quantity per unit, and excipients [1,2,31].

Pharmacopoeial control is centred on identity and hederacoside C content. Published research methods add α-hederin, hederasaponin B, chlorogenic acid, rutin, and other markers. Comparative profiling has been used to examine raw material, intermediate extracts, and finished products [3,5,29,30].

The medicinal substance cannot be described adequately by the species name alone. The EU monograph distinguishes dry extracts prepared with approximately 24–30%, 40%, or 60% ethanol, a 1:1 liquid extract prepared with 70% ethanol, and a soft extract prepared with 50% ethanol and propylene glycol. Their drug–extract ratios range from 1:1 to 8:1. These variables change the mass of native leaf represented by one milligram of extract and the proportion of saponins, phenolics, and other co-extractives carried into the finished dosage form. Evidence transfer therefore requires the same herbal preparation or data establishing chemical and pharmaceutical comparability [1,2,3].

Hederacoside C serves as the principal release marker and is required at not less than 3.0% in the dried herbal drug. α-Hederin deserves additional measurement in extracts intended for mechanistic or clinical comparison, since it can occur in the plant, arise during manufacture, or form through hydrolysis of hederacoside C. Published HPLC-DAD and LC–MS/MS methods can measure these saponins together with hederacoside D, rutin, chlorogenic acid, and other phenolic markers. Multi-analyte fingerprints are consequently available for raw material, extract, stability, and finished product comparisons even though they are not yet routinely linked to clinical bridging criteria (Table 5) [4,5,29,30].

Evidence synthesis. The saponin-dominated constituent profile, the role of hederacoside C as the pharmacopoeial marker, and the availability of published multi-analyte analytical methods can be considered established [3,4,5,29,30]. The magnitude and clinical relevance of batch-to-batch and product-to-product variability remain insufficiently characterised, and routine quantification of α-hederin is not currently a pharmacopoeial requirement despite its prominence in mechanistic studies [3,5,6,29,30,31]. The principal translational limitation is preparation non-equivalence: the extract categories recognised in the EU monograph differ in drug–extract ratio and extraction solvent, and evidence obtained with one preparation should not be assumed to be transferable to another without supporting pharmaceutical and chemical comparability data [1,2,29,30,31].

## 5. Traditional and Contemporary Medicinal Uses

Historical European sources describe the use of ivy leaves and other plant parts for respiratory catarrh, cough, inflammatory complaints, wounds, burns, rheumatic conditions, and parasites. Respiratory use became the principal modern medicinal application. ESCOP and the German Commission E described ivy leaf for catarrhal or inflammatory bronchial complaints before the adoption of the current European Union monograph [19,36,37].

The current EU therapeutic indication is use as an expectorant in productive cough. Clinical studies have enrolled patients with acute cough, acute bronchitis, common cold-associated cough, mixed acute and chronic inflammatory bronchial disorders, and paediatric respiratory illness. Small studies have also evaluated add-on use in children with bronchial asthma [1,10,12,13,15].

Products include syrups, oral drops, film-coated tablets, effervescent, or other solid forms. Studies have used different extracts and formulations, including the named dry extract EA 575 and other specified or incompletely specified preparations. Combination products containing thyme, primrose, or other herbs have also been studied; the present clinical efficacy summary separates these from ivy mono-preparations [2,11,18,38].

Productive cough is the indication stated in the EU monograph. The clinical programme includes adults with acute cough, adults with acute bronchitis defined by a Bronchitis Severity Score, mixed adult and paediatric bronchitis cohorts, and schoolchildren with acute bronchitis. Treatment duration in the principal modern studies was seven days, with some follow-up assessments through day 14 [1,10,12,13,18,39].

Acute bronchitis studies used placebo, another ivy extract, acetylcysteine, or herbal combinations as comparators. The placebo-controlled trials tested EA 575; the 590-patient trial compared two ivy extracts; the non-interventional study compared ivy syrup with acetylcysteine; and the 2025 trial compared EA 575 with ivy/thyme and thyme/primrose [11,13,18,40].

Some publications enrolled patients under the labels acute respiratory tract infection, common cold, or cold-associated cough rather than acute bronchitis. The randomised acute cough trial, the tablet observational study, systematic reviews, and the paediatric prescription database study fall within this group. Direct virological outcomes were not measured in the principal ivy mono-preparation trials [12,16,23,38,41].

The 9657-patient postmarketing study included acute and chronic inflammatory bronchial diagnoses. Older studies and product-specific reviews also report chronic respiratory use. The present EU monograph is worded for productive cough and does not list chronic obstructive pulmonary disease or asthma as separate indications [1,10,21].

Asthma studies used ivy as add-on treatment. Hofmann et al. summarised older randomised paediatric investigations, and Zeil et al. conducted a 30-patient double-blind crossover trial in children receiving inhaled corticosteroids. Lung function outcomes rather than acute productive cough outcomes were assessed [15,42].

EMA preparation categories cover dry, liquid, and soft extracts. Clinical publications describe syrups, drops, film-coated tablets, and other solid or liquid dosage forms. The administered dose is expressed as the quantity of the specified extract, and formulation excipients vary [1,2,38,39].

Evidence synthesis. Historical respiratory use and its continuity into the modern expectorant indication are well documented [19,36,37]. The current EU monograph recognises specified ivy leaf preparations under well-established use as expectorants in productive cough. Broader historical uses and studies conducted in asthma, chronic bronchial disorders, or broadly defined respiratory infections are not equivalent to this indication and do not establish additional monograph uses [1,2,10,15,21,23,42]. Uncertainty remains regarding the applicability of older chronic-bronchial evidence and legacy product indications to the preparations currently defined in the EU monograph [1,2,10,21]. The principal limitation for clinical interpretation is product specificity: evidence obtained with a particular extract and formulation should not be generalised to “ivy” as a therapeutic class. Differences in extraction, dosage form, patient age, treatment duration, and diagnostic definition further limit cross-study extrapolation; conclusions should remain linked to the exact preparation, population, and respiratory condition examined [1,2,10,11,12,13,14,15,21,38,39,40,41,42].

## 6. Pharmacodynamics

### 6.1. Respiratory Pharmacodynamics

Secretolytic activity of *H. helix* has been described in regulatory reports, older pharmacological studies, and recent animal experiments. Proposed measurements include surfactant secretion, tracheobronchial secretion, mucus transport, and clinical expectoration (Table 6) [2,21].

Hufnagel et al. administered EA 575 orally in guinea pig and mouse models. In a mouse phenol red secretion assay, doses within the tested range increased recovery of dye from the tracheobronchial tract, with a reported maximum increase of 38.9%. In guinea pigs, the same programme evaluated cough events and inflammatory cells after bleomycin or citric acid challenge. These experiments supplied direct in vivo measurements after oral administration of a defined extract [7].

**Figure 3:**
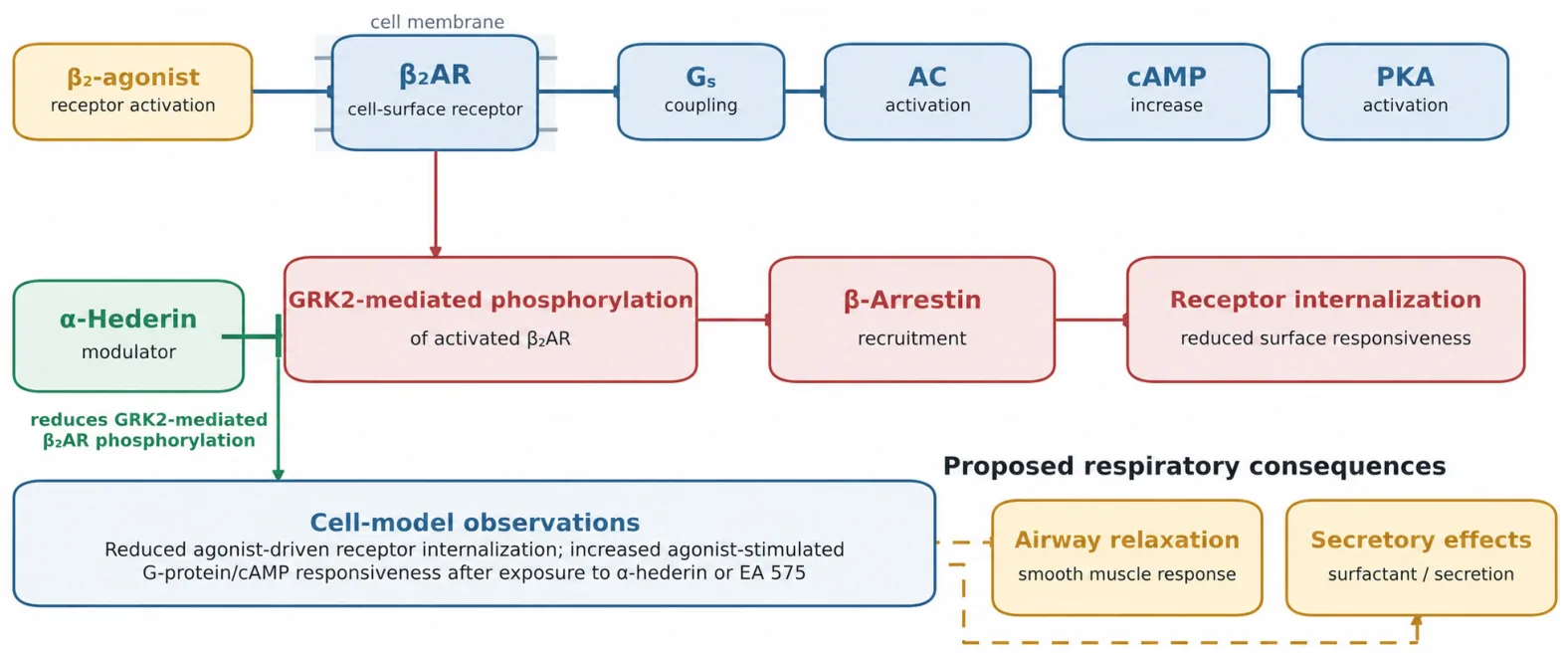
β_2_-adrenergic signalling and the proposed site of action of α-hederin. Agonist binding to the β_2_-adrenergic receptor (β_2_AR) activates the stimulatory G protein (Gs), adenylyl cyclase (AC), cyclic AMP (cAMP) formation, and protein kinase A (PKA). Activated receptors are phosphorylated by G protein-coupled receptor kinase 2 (GRK2), recruiting β-arrestin and promoting receptor internalisation. α-Hederin reduces GRK2-mediated β_2_AR phosphorylation; in cell models, exposure to α-hederin or the ivy leaf dry extract EA 575 reduced agonist-driven receptor internalisation and increased agonist-stimulated G-protein/cAMP responsiveness. The respiratory consequences (airway smooth muscle relaxation; surfactant/secretory effects) are proposed on this basis. Cellular effects were demonstrated at micromolar concentrations in vitro, whereas measured lung tissue exposure after oral dosing is substantially lower; clinical relevance is therefore inferred rather than directly demonstrated.

β2-Adrenergic signalling has been proposed to increase surfactant production in type II pneumocytes. Experiments with α-hederin and ivy extract measured receptor internalisation, cAMP production, and downstream signalling rather than surfactant release in patients. The secretolytic mechanism is therefore represented by linked cellular and animal endpoints rather than a completed human pharmacodynamic study (Figure 3) [2,6,43,44].

The early antispasmodic work used isolated guinea pig ileum stimulated with acetylcholine. Trute et al. compared a dry ivy extract with purified constituents and expressed activity as papaverine equivalents. Reported values were approximately 55 for α-hederin, 49 for hederagenin, 54 for quercetin, 143 for kaempferol, and 22 for dicaffeoylquinic acid, while hederacoside C showed much lower activity, near 6 [45].

In a conscious guinea pig model, oral ethanolic ivy extract at 50 mg/kg reduced compressed air bronchoconstriction induced by ovalbumin or platelet-activating factor by 57% and 43%, respectively. The result provided whole-animal evidence for bronchospasmolytic activity after enteral administration, although the extract was not described with the full chemical fingerprint now expected for product bridging. Airway smooth muscle experiments supplied a more specific mechanistic sequence through α-hederin pretreatment and enhanced β-adrenoceptor-mediated relaxation [2,46].

The receptor trafficking experiments quantified this sequence. In HEK293 cells, 1 μM α-hederin for 24 h inhibited agonist-driven internalisation, whereas hederacoside C and hederagenin were inactive under the same conditions. In A549 cells, total fluorescent noradrenaline-analogue binding increased from 33.0 ± 6.8% to 41.2 ± 4.6%, and the reported dissociation constant decreased from 36.1 ± 9.2 to 24.3 ± 11.1 nM. In human airway smooth muscle cells, stimulated cAMP increased by 13.5 ± 7.0% [6,43].

The translational exposure comparison remains incomplete. The EMA assessment cited an older radiolabel study that found a maximum lung tissue concentration of approximately 0.018 μg/g at 24 h, equivalent to about 0.024 nmol/g, below the 0.5–1 μM concentrations used in key cell experiments. The recent animal work with oral EA 575 adds functional endpoints: selected regimens reduced cough events by up to 56%, airway neutrophils by up to 70.9%, and increased phenol red recovery from the tracheobronchial tract by up to 38.9% [2,7].

In the 2025 preclinical study, guinea pigs received oral EA 575 in bleomycin-associated and citric acid cough models. Cough events were counted after challenge, and reductions of up to 56% were reported in selected dosing conditions. In the inflammatory model, airway neutrophils were reduced by up to 70.9%. The tested daily doses ranged from approximately 43 to 430.5 mg/kg [7].

Earlier isolated tissue studies examined contractions in guinea pig ileum and airway preparations. Trute et al. tested a dry ivy extract and isolated antispasmodic constituents; Capasso et al. assessed flavonoid-related inhibition of agonist-induced contraction. Wolf et al. used airway smooth muscle preparations pretreated with α-hederin and measured enhanced β-adrenoceptor-mediated relaxation [45,46,47].

A paediatric crossover study in controlled allergic asthma measured spirometric variables during add-on ivy therapy. The main FEV1 and mid-expiratory flow outcomes did not show a clear significant difference, while selected secondary flow measurements changed. No adult clinical bronchodilator study with acute reversibility testing or comparison with an inhaled β2 agonist was identified [15].

Hegener et al. established live-cell methods for monitoring β2-adrenergic receptor–ligand complexes. Sieben et al. subsequently preincubated β2 receptor-expressing HEK293 cells with α-hederin, hederacoside C, or hederagenin and measured agonist-induced receptor internalisation. α-Hederin reduced internalisation; hederacoside C and hederagenin did not produce the same effect under the tested conditions [6,48].

Greunke et al. studied human airway smooth muscle cells and measured β2-agonist-stimulated cAMP after exposure to major ivy extract constituents. α-Hederin increased responsiveness in the experimental system. Wolf et al. measured greater β-adrenoceptor-mediated relaxation after α-hederin pretreatment in airway smooth muscle [43,46].

Schulte-Michels et al. examined receptor phosphorylation and reported inhibition of G protein-coupled receptor kinase 2-mediated β2 receptor phosphorylation by α-hederin. Meurer et al. tested EA 575 in signalling assays and reported increased G-protein/cAMP signalling with reduced β-arrestin recruitment under stimulated conditions, described as biased β2-adrenergic signalling [44,49].

**Table 6:** Primary nonclinical respiratory studies of *Hedera helix* extracts and constituents.

Test Material	Model/Exposure	Endpoints	Reported Findings	Reference
Dry ivy extract and isolated constituents	Isolated smooth muscle preparations; in vitro	Agonist-induced contraction	Papaverine-equivalent activity: α-hederin 55, hederagenin 49, quercetin 54, kaempferol 143, dicaffeoylquinic acid 22, hederacoside C ~6	[45]
β2 receptor ligands/methodological system	Living receptor-expressing cells	Receptor–ligand complex dynamics	Established the live-cell framework used in later ivy studies	[48]
α-Hederin, hederacoside C, hederagenin	HEK293 β2 receptor cells; prolonged preincubation	Agonist-induced receptor internalisation	1 μM α-hederin reduced receptor internalisation; ligand binding rose from 33.0 ± 6.8% to 41.2 ± 4.6%; KD fell from 36.1 ± 9.2 to 24.3 ± 11.1 nM	[6]
α-Hederin	Airway smooth muscle preparations	β-adrenoceptor-mediated relaxation	Pretreatment increased relaxation during β-adrenergic stimulation	[46]
Major ivy extract constituents	Human airway smooth muscle cells	β2-agonist-stimulated cAMP	α-Hederin increased β2 responsiveness	[43]
α-Hederin	β2 receptor cell model	PKA/GRK2-mediated phosphorylation	GRK2-mediated receptor phosphorylation was reduced	[49]
EA 575	β2 signalling assays	cAMP and β-arrestin recruitment	Signalling pattern favoured G-protein/cAMP response	[44]
Oral EA 575	Guinea pig cough and mouse phenol red models; ~43–430.5 mg/kg/day	Cough events, neutrophils, tracheobronchial dye secretion	Up to 56% fewer cough events, 70.9% fewer neutrophils, and 38.9% greater dye secretion in selected conditions	[7]
Ethanolic ivy extract	Conscious guinea pigs; oral 50 mg/kg; ovalbumin or PAF challenge	Compressed air bronchoconstriction	Inhibition of 57% after ovalbumin and 43% after PAF challenge	[2]

cAMP, cyclic AMP; GRK2, G protein-coupled receptor kinase 2; PAF, platelet-activating factor; PKA, protein kinase A. Percentages are maxima reported within the 2025 experimental programme and do not represent human effect sizes.

#### Translational Limitations of the Proposed β_2_-Adrenergic Mechanism

The cellular evidence supporting the proposed β_2_-adrenergic mechanism carries inherent model limitations. The receptor trafficking experiments used HEK293 cells heterologously expressing β_2_-adrenergic receptor–GFP fusion proteins, A549 cells as a carcinoma-derived alveolar epithelial surrogate, and cultured human airway smooth muscle cells [6,43,48,49]. Overexpressed and fluorophore-tagged receptors may not reproduce the density, trafficking kinetics, or regulatory context of native receptors. Immortalised or carcinoma-derived cell lines differ from differentiated airway epithelium, while monolayer cultures lack the air–liquid interface, mucus layer, ciliary activity, and neurohumoral, vascular and immune inputs that contribute to integrated airway function. The principal receptor internalisation protocol also used continuous 24 h preincubation with α-hederin, an exposure pattern that does not reproduce the intermittent systemic exposure expected after oral administration. Isolated tissue and animal experiments provide organ-level and whole-animal endpoints but retain species-related limitations and, particularly in older studies, incomplete extract characterisation [7,45,46,47]. These models establish cellular and physiological plausibility but do not demonstrate that the same pathway operates during therapeutic use in humans.

A quantitative gap separates the experimental concentrations from documented clinical exposure. The principal cellular effects were obtained with approximately 0.5–1 μM α-hederin under prolonged incubation conditions [6,43,49]. In contrast, the available human observations detected no or only trace α-hederin in blood; in one investigation, concentrations of 1.39–1.51 nmol/L were detected in only two of sixteen volunteers. These exploratory studies did not generate validated concentration–time profiles or conventional pharmacokinetic parameters [2]. Rat studies reported absolute oral bioavailability of approximately 0.14% for α-hederin [50]. The maximum lung tissue concentration summarised from the older radiolabel study was approximately 0.018 μg/g, equivalent to approximately 0.024 nmol/g at 24 h, which is numerically more than one order of magnitude below the concentrations used in the principal cellular experiments [2]. This comparison remains approximate: total radiolabel in animal lung tissue is not equivalent to the unbound concentration of unchanged α-hederin at a human airway receptor. The free fraction at the receptor, local airway concentrations after repeated dosing, and the contribution of unmeasured metabolites remain unknown. It has therefore not been established that labelled oral treatment produces target site exposure corresponding to the concentrations required for the reported cellular effects.

No confirmatory human pharmacodynamic biomarker of β_2_-adrenergic pathway engagement has been reported. Clinical studies principally measured cough symptoms, Bronchitis Severity Score, global clinical improvement and, in a limited number of asthma studies, spirometric or airway resistance variables [10,11,12,13,14,15,16,22]. No trial measured β_2_ receptor surface density or internalisation, airway cAMP generation, GRK2- or PKA-dependent receptor phosphorylation, β-arrestin recruitment, or an exposure–response relationship for these endpoints. Objective downstream measurements—including bronchodilator responsiveness benchmarked against an inhaled β_2_ agonist, surfactant-associated components in respiratory samples, sputum rheology, and mucociliary clearance—were likewise not incorporated into the clinical programme. In the paediatric asthma crossover trial, the primary lung function comparison did not demonstrate a clear significant difference [15]. Symptomatic efficacy and isolated spirometric findings are therefore compatible with, but do not verify, the proposed receptor mechanism. The β_2_-adrenergic model should consequently be presented as a biologically plausible mechanism supported by nonclinical experiments but not yet confirmed in therapeutic exposure in humans. Biomarker-anchored studies linking validated human exposure to prespecified β_2_-pathway and downstream respiratory endpoints are identified as a research priority in Section 12.

### 6.2. Secondary and Supportive Pharmacology

Süleyman et al. tested an ivy preparation in rat models of acute and chronic inflammation and measured reductions in inflammatory responses. Gepdiremen et al. administered α-hederin, hederasaponin C, and hederacolchisides E and F orally in carrageenan-induced rat paw edema and reported activity for the isolated saponins. Lutsenko et al. examined ivy leaf extracts in experimental lung and bronchial models and reported anti-inflammatory and antimicrobial outcomes [51,52,53].

Schulte-Michels et al. exposed murine macrophages to EA 575 before lipopolysaccharide stimulation and measured interleukin-6 release; the extract reduced the measured response at 80–400 μg/mL. Meurer et al. studied Calu-3 airway epithelial cells and reported inhibition of adenosine A2B receptor signalling and inflammatory output after extract exposure [54,55].

Čolić et al. treated human monocyte-derived dendritic cells with EA 575 and assessed differentiation, maturation markers, cytokine production, and subsequent T-cell responses. Changes were reported across the dendritic cell and co-culture endpoints [56].

The classical inflammation studies also provide dose and comparator information. In carrageenan-induced acute paw edema, a crude saponin extract at 100–200 mg/kg produced a maximum reported inhibition of 77%, compared with 89.2% for indomethacin. In the cotton pellet granuloma model, the purified saponin extract, crude saponin extract, and indomethacin produced reported inhibitions of 60%, 49%, and 66%, respectively. Oral α-hederin and hederacoside C were subsequently active in the second phase of carrageenan edema. These studies support anti-inflammatory activity of ivy saponins in rodents, with no direct clinical inflammatory endpoint [51,52].

Secondary pharmacology covers a broad but uneven dataset. Ivy saponins inhibited Gram-positive organisms at reported minimum inhibitory concentrations of 0.312–1.25 mg/mL and Gram-negative organisms at 1.25–5 mg/mL. α-Hederin inhibited several *Candida* and dermatophyte species within a reported range of 5–100 μg/mL and altered the *C. albicans* cell envelope on electron microscopy. Separate experiments described enterovirus, influenza screening, Leishmania, helminth, mollusc, antioxidant, antinociceptive, gastric-motor, and hepatic effects. These experiments used different plant parts, fractions, purified saponins, routes, and concentrations [57,58,59,60,61,62,63,64,65,66]. Table 7 records them as nonclinical activities rather than respiratory indications.

Akhtar et al. used hederacoside C in RAW264.7 cells and in a mouse model of *Staphylococcus aureus*-induced acute lung inflammation. The study measured pulmonary edema, white cell counts, wet-to-dry ratio, myeloperoxidase activity, and TLR-related signalling; hederacoside C reduced the reported inflammatory measures. Shokry et al. used a phenolic ivy fraction in lipopolysaccharide-induced mouse lung injury and reported reductions in histological injury, oxidative stress, and inflammatory markers [32,67].

Gülçin et al. tested α-hederin, hederasaponin C, and hederacolchisides E and F in antioxidant assays and reported radical scavenging and related activity. Geographic profiling studies measured antioxidant capacity together with phenolic acid composition. The phenolic fraction lung injury study also measured oxidative endpoints in vivo [28,32,57].

Elias et al. tested saponins from *H. helix* and other plants in mutagenicity-related assays. Amara-Mokrane et al. assessed α-hederin in doxorubicin-exposed human lymphocytes and measured micronuclei. Mba Gachou et al. tested α-hederin against hydrogen peroxide-induced DNA damage in HepG2 cells using the comet assay. Villani et al. also reported in vitro antimutagenic testing of α-hederin [68,69,70,71].

Early antimicrobial screening identified antibacterial activity in ivy saponin-containing preparations. Cioaca et al. reported antibacterial effects of ivy saponins, and Ieven et al. included *Hedera* material in a broader medicinal plant screen. The tested organisms, extract definitions, and concentrations varied between reports [58,72].

Favel et al. tested triterpenoid saponins against fungi and reported antifungal activity. Moulin-Traffort et al. used ultrastructural methods to examine *C. albicans* after α-hederin exposure and described membrane and intracellular changes [59,60].

Song et al. studied hederasaponin B against enterovirus 71 subgenotypes C3 and C4a in cell culture and reported inhibition of viral activity. Rao et al. reported earlier antiviral testing of triterpenoid saponins. A rapid review of human viral respiratory evidence did not identify clinical trials demonstrating a direct antiviral effect of ivy preparations [23,61,73].

Hostettmann and colleagues measured molluscicidal activity of ivy saponins. Majester-Savornin et al. reported leishmanicidal activity. Eguale et al. tested aqueous and hydroalcoholic ivy extracts against *Haemonchus contortus* in vitro and in vivo, and Urban et al. included ivy in in vitro anthelmintic screening [62,63,64,74,75].

Liu et al. administered α-hederin in experimental models and measured suppression of hepatic cytochrome P450 pathways. Jeong and Park studied carbon tetrachloride-induced hepatotoxicity in mice and reported reduced injury together with lower CYP2E1 expression. Jeong separately reported suppression of constitutive and inducible cytochrome P450 gene expression in mice [65,76,77].

Mendel et al. exposed isolated rat stomach strips to whole ivy extract and selected constituents and measured motor activity. Facino et al. examined elastase and hyaluronidase inhibition by saponins and sapogenins from ivy and other medicinal plants [66,78].

These experiments constitute secondary pharmacology rather than respiratory clinical studies. Published broad reviews additionally catalogue analgesic, antinociceptive, vascular, molluscicidal, cytotoxic, and other exploratory activities (Table 7). The present review lists these activities only where a primary study could be connected to an identified ivy material or constituent. No clinical indication outside of productive cough was assigned on the basis of these experiments.

**Table 7:** Overview of secondary nonclinical pharmacology.

Activity	Representative Material/Study	Experimental System	Reported Outcome	Evidence Type	Reference
Anti-inflammatory	Ivy extract and isolated saponins	Rat edema/inflammation models	Reduced inflammatory responses	In vivo	[51,52]
Airway inflammation	Hederacoside C	S. aureus lung inflammation in mice; RAW264.7 cells	Reduced edema, cells, MPO, and TLR-pathway measures	In vivo/in vitro	[57]
Macrophage/A2B signalling	EA 575	Murine macrophages and Calu-3 cells	Reduced IL-6 or A2B-pathway signaling	In vitro	[54,55]
Dendritic cell effects	EA 575	Human monocyte-derived dendritic cells and T-cell co-culture	Altered differentiation, maturation, cytokines, and T-cell responses	In vitro	[56]
Antioxidant/cytoprotective	Ivy saponins and α-hederin	Chemical assays, lymphocytes, HepG2 cells	Antioxidant measures and reduced induced DNA damage	In vitro	[67,69,70]
Antibacterial/antifungal	Saponins and α-hederin	Microbial growth and ultrastructure assays	Growth inhibition and Candida structural changes	In vitro	[58,59,60]
Antiviral	Hederasaponin B	Enterovirus 71 cell culture	Reduced viral activity in tested subgenotypes	In vitro	[61]
Antiparasitic	Extracts/saponins	Leishmania and helminth models	Reduced viability or motility	In vitro/in vivo	[63,64,75]
Hepatic/CYP	α-Hederin	Rodent hepatic injury and enzyme expression models	CYP suppression and reduced CCl4 injury	In vivo	[65,76,77]
Gastrointestinal	Whole extract and constituents	Isolated rat stomach strips	Changes in motor activity	Ex vivo	[66]

A2B, adenosine A2B receptor; CCl4, carbon tetrachloride; CYP, cytochrome P450; IL-6, interleukin-6; MPO, myeloperoxidase; TLR, Toll-like receptor.

### 6.3. Evidence Map by Test Material

Isolated constituent studies most frequently used α-hederin, hederacoside C, hederagenin, hederasaponin C, hederasaponin B, or related hederacolchisides. α-Hederin was used in receptor trafficking, airway relaxation, cytochrome P450, genotoxicity, antifungal, and developmental studies. Hederacoside C was used as an analyte in pharmacokinetic work and as the intervention in the *S. aureus* lung inflammation study. Hederasaponin B was used in the enterovirus study [6,35,46,60,61,65,67,69].

Defined complete extract studies include the EA 575 β2 signalling, macrophage, A2B-receptor, dendritic cell, and 2025 animal cough/secretion experiments. The same named extract appears in the modern placebo-controlled adult clinical programme and the 2025 head-to-head trial [7,12,13,18,44,54,55,56].

Other studies used a dry ivy extract, aqueous or hydroalcoholic extracts, phenolic fractions, crude leaf extracts, or incompletely specified pharmaceutical preparations. The anti-inflammatory rat studies, phenolic fraction lung study, gastrointestinal strip study, and several antimicrobial or antiparasitic experiments fall within this group [32,51,58,64,66].

Exposure reporting differed between experiments. Cell studies generally reported concentrations and incubation time; animal studies reported dose and route; isolated tissue studies reported bath concentration; and clinical studies reported formulation and dosing schedule.

Evidence synthesis. Within the experimental systems examined, the respiratory pharmacology is directionally consistent. α-Hederin modulates agonist-induced β2-adrenergic receptor internalisation and desensitisation-related signalling in cellular and airway smooth muscle models; EA 575 demonstrates anti-inflammatory and immunomodulatory activity in vitro; and oral EA 575 produces cough reduction and increased tracheobronchial secretion in animal models [6,7,43,44,45,46,47,48,49,51,52,53,54,55,56]. These endpoints are complementary rather than interchangeable, and the secondary antimicrobial and systemic activities reported for ivy constituents should not be interpreted as evidence of respiratory clinical efficacy. No directly contradictory mechanistic findings were identified. The unresolved question concerns the relative contribution of β2 receptor-mediated surfactant regulation, local gastrointestinal or reflex pathways, anti-inflammatory activity, and other mechanisms to the clinical expectorant effect [2,7,21,43,44,66]. Much of the receptor and signalling evidence originates from interconnected research groups, and independent replication remains limited. The principal translational constraints—artificiality of the cellular models, incomplete characterisation of some older extracts, the disparity between experimental concentrations and documented clinical exposure, and the absence of confirmatory human target engagement biomarkers—are examined in Section Translational Limitations of the Proposed β2-Adrenergic Mechanism [2,6,7,43,44,45,46,47,48,49].

## 7. Pharmacokinetics

### 7.1. Preclinical Disposition and ADME

Kim et al. developed a plasma assay and studied hederacoside C in rats after administration of AG NPP709 and the reference compound. Plasma concentration–time profiles were generated and used to estimate pharmacokinetic parameters. The study demonstrated measurable systemic exposure and dose-related disposition in rats. Kim et al. administered hederacoside C intravenously at 3, 12.5, and 25 mg/kg. Area under the curve increased proportionally over that range, while half-life, clearance, steady-state distribution volume, and mean residence time were broadly dose-independent. Urinary recovery accounted for 16.8–31.9% of dose and gastrointestinal recovery for less than 0.712%, indicating an additional nonrenal elimination component. After oral pure compound at 12.5–50 mg/kg, absolute bioavailability was 0.184% at 12.5 mg/kg and 0.250% at 25 mg/kg. Recovery from the gastrointestinal tract included hederacoside C and the downstream compounds α-hederin and hederagenin [8].

Yu et al. measured hederacoside C, hederacoside D, and α-hederin in rat plasma after administration of *H. helix* material. The three analytes showed different concentration–time profiles, supporting simultaneous measurement of parent saponins and related metabolites [9].

When an ivy extract was given at hederacoside C-equivalent oral doses of 12.5, 25, and 50 mg/kg, bioavailability was 0.186% at 12.5 mg/kg and 0.118% at 25 mg/kg. At 50 mg/kg, dose-normalised area under the curve and peak concentration rose disproportionately, and renal clearance was slower. Across the intravenous experiments, total clearance was approximately 1.46–2.08 mL/min/kg and steady-state distribution volume was approximately 138–222 mL/kg. The matrix-dependent and nonlinear observations show that the isolated marker and the complete extract cannot be assumed to have identical disposition [8].

Li et al. quantified α-hederin in rat plasma after intravenous and oral administration. The reported absolute oral bioavailability was approximately 0.14%, and the terminal half-life after oral dosing was about 2.67 h in the tested experiment [50].

The available human observations were analytical pilot studies rather than complete pharmacokinetic trials. After a single 1 g dose of a dry extract containing 6.5% hederacoside C and 4.0% α-hederin in one volunteer, α-hederin was not detected. Four volunteers then received 130 mg of the extract twice daily for seven days; three had small peaks near the detection limit, with estimated whole-blood concentrations of 0.8, 0.6, 0.5, and 0 μg/mL across the four participants. In another 16-volunteer study using 130 mg daily, α-hederin was detected in only two participants at 1.39–1.51 nmol/L plasma. No validated concentration–time parameters, bioavailability estimate, metabolite profile, or exposure–response analysis resulted [2].

Rehman et al. validated an UHPLC–MS/MS method for hederacoside C in rat plasma. The assay provided the sensitivity and selectivity required for preclinical concentration measurement and was applied to pharmacokinetic samples [79].

The rat studies show limited and analyte-dependent oral exposure. Hederacoside C is a highly glycosylated saponin; α-hederin is less glycosylated; and hederagenin is the aglycone. Sequential deglycosylation from hederacoside C to α-hederin and hederagenin has been proposed on the basis of chemical structure, extract processing, and animal analyte profiles [2,8,9,50].

Oral and intravenous comparison in the α-hederin study enabled calculation of absolute bioavailability. The reported value of approximately 0.14% applies to isolated α-hederin in rats under that protocol. The hederacoside C studies used different test material and analytical methods and should be reported with their own dosing conditions [8,9,50].

Quantitative pulmonary distribution was not established in the modern studies. The EMA assessment summarises older distribution information and notes that measured tissue concentrations were below concentrations used in some pharmacodynamic experiments [2].

The available publications principally report plasma concentrations. Free versus protein-bound fractions, epithelial lining fluid concentrations, bronchial secretion concentrations, and distribution to airway smooth muscle were not measured [8,9,50].

Deglycosylation is the main proposed transformation of hederacoside C. α-Hederin represents a partially deglycosylated product, and hederagenin represents the aglycone. The relative contribution of manufacturing conversion, intestinal enzymes, gut microbiota, and systemic metabolism has not been quantified in humans. Phase II conjugation of the aglycone has been proposed but was not completely characterised in the cited ivy studies [2,4,9].

The rat studies estimated terminal concentration–time parameters for measured analytes, but a complete balance of renal, biliary, and faecal elimination was not reported for a finished ivy extract. The α-hederin study reported an oral terminal half-life of about 2.67 h under its experimental conditions [8,9,50].

### 7.2. Human Exposure and Interaction Evidence

No published therapeutic-dose human pharmacokinetic study measuring hederacoside C, α-hederin, hederagenin, or related metabolites was identified. Clinical trials reported administered extract volumes or product doses and clinical outcomes without collection of plasma concentration–time data. Human bioavailability, food effects, repeated-dose accumulation, metabolic variability, and pulmonary exposure therefore remain unmeasured [2,16].

Rehman et al. incubated ivy extracts with human liver microsomes and measured CYP2C8, CYP2C19, and CYP2D6 activity. Concentration-dependent inhibition was reported, with time-dependent inhibition for CYP2C8 and CYP2C19. No clinical interaction trial was identified, and the EMA monograph lists no established clinical interactions [1,2,80].

Interaction evidence likewise remains exposure-limited. Subcutaneous α-hederin at 10–30 μmol/kg for three days reduced hepatic cytochrome P450 content and selected enzyme activities in mice by approximately 30–50%; other experiments used 8–80 mg/kg and reported dose- and time-related suppression of CYP1A1/2 and CYP2E1 measures. The route bypassed intestinal absorption, and the lowest experimental systemic dose was estimated in the regulatory assessment to exceed the usual oral human exposure substantially. Human liver microsome studies found time-dependent inhibition of CYP2C8, CYP2C19, and CYP2D6 by ivy extracts, but no clinical interaction study has quantified the in vivo magnitude [2,65,77,80].

This absence of human pharmacokinetic characterisation should be regarded as the principal limitation of the current evidence base. Without therapeutic-dose concentration–time data for hederacoside C, α-hederin, hederagenin, and their conjugates, systemic exposure cannot be linked to the receptor-level mechanisms observed in vitro, safety margins for membrane-related and interaction-related findings cannot be quantified, and dose selection across age groups remains supported by clinical outcome data alone. A single- and repeated-dose pharmacokinetic study at labelled doses, using validated LC–MS/MS assays and documented extract batches, would address this gap directly (Section 12).

The available pharmacokinetic and interaction studies of ivy constituents and extracts are summarised in Table 8.

The modern pharmacokinetic dataset contains several rat plasma studies and one human liver microsome interaction study. It does not contain a therapeutic-dose human concentration–time study, pulmonary microdialysis or epithelial lining fluid study, complete extract mass balance study, or clinical exposure–response analysis [2,8,9,50,80].

The measured analytes differ across publications. Hederacoside C is present in the Kim and Rehman’s work; hederacoside C, hederacoside D, and α-hederin are included in the Yu study; and isolated α-hederin is measured in the Li study. Hederagenin and phase II conjugates were not comprehensively measured in the cited studies [8,9,50,79].

Clinical trials provide dosing and outcome information but no plasma sampling. As a result, interindividual pharmacokinetic variability by age, food intake, gastrointestinal transit, gut microbiota, formulation, liver function, or kidney function has not been described [2,12,13,16].

Evidence synthesis. Rat studies establish that hederacoside C and α-hederin can reach systemic circulation after oral administration, although the reported absolute oral bioavailability estimates are very low. Disposition is dependent on the analyte, administered material, dose, and analytical method, while the available findings are compatible with sequential deglycosylation of hederacoside C to α-hederin and subsequently to hederagenin [8,9,50,79]. Numerical differences among studies therefore reflect differences in experimental design and test material rather than directly contradictory pharmacokinetic findings. Human disposition remains largely uncharacterised: no validated therapeutic dose concentration–time profile, comprehensive metabolite or excretion balance, unbound plasma fraction, airway distribution measurement, or exposure–response relationship is available [2,8,9,50,79,80]. This represents one of the principal cross-domain limitations of the evidence base. Without reliable patient exposure data, it is not possible to determine whether concentrations associated with β2-adrenergic effects in vitro are achieved during labelled oral treatment, calculate quantitative margins against the toxicological findings, or establish human dose proportionality and pulmonary target exposure [2,6,8,9,50,79,80].

## 8. Toxicology

### 8.1. Acute and Repeated-Dose Toxicity

The EMA assessment summarises acute and repeated-dose studies of ivy extracts and constituents. Acute oral toxicity was low in the reported experiments relative to therapeutic dosing. Gastrointestinal and membrane-related effects occurred at high exposure. In a 90-day rat study summarised by EMA, a high oral extract dose was associated with haemolytic findings. Full modern toxicokinetic data were not available [2].

The acute dataset includes several extract and saponin preparations. Oral median lethal doses in mice were reported above 3 g/kg for multiple ivy extracts; rats given a dry extract at up to 4.1 g/kg had no deaths during 72 h, with diarrhoea as the main observation. Oral LD50 values for a 60% saponin mixture, α-hederin, and hederacoside C were reported above 4 g/kg, whereas intraperitoneal LD50 values were lower, at approximately 1.8 g/kg for α-hederin and 2.3 g/kg for the saponin mixture. The route contrast is consistent with limited gastrointestinal uptake of these saponins [2].

Repeated oral administration was examined in older rat studies. A dry extract at 1.5 g/kg/day for 100 days was reported without treatment-related toxicity. A hydroethanolic dry extract at 4 g/kg/day for 90 days produced haemolytic findings. The high doses provide a qualitative hazard boundary, but published study documentation does not supply the full clinical pathology, histopathology, toxicokinetic, recovery, and Good Laboratory Practice detail expected in a modern repeated-dose programme [2].

Triterpenoid saponins are amphiphilic, membrane-active constituents that can self-aggregate and interact with cholesterol and phospholipids in cellular membranes. At sufficiently high free concentrations, membrane-active saponins alter lipid bilayer organisation, modify membrane cholesterol distribution, and increase membrane permeability. The formation of pores or larger membrane defects permits ionic flux, osmotic swelling, and haemoglobin release, ultimately resulting in erythrocyte lysis. In a cellular membrane model, α-hederin displayed membrane toxicity associated with a reduction in membrane cholesterol, whereas hederacoside C was substantially less membrane-active under the tested conditions. These findings support a structure- and concentration-dependent mechanism rather than a uniform class effect across all ivy saponins [81,82].

Membrane perturbation is not restricted to erythrocytes. In nucleated cells, sufficiently high free concentrations of α-hederin may disturb transmembrane ion gradients, calcium homeostasis, membrane-associated signalling, and mitochondrial integrity, ultimately promoting apoptotic or lytic cell death. The balance between membrane permeabilisation and intracellular stress depends on concentration, exposure duration, membrane composition, and cholesterol content. This mechanism provides a plausible explanation for cytotoxic effects observed under direct cellular exposure but does not establish toxicity at the substantially lower systemic concentrations expected after therapeutic oral administration [81,82].

The route of administration and resulting systemic exposure are decisive for the clinical interpretation of this intrinsic haemolytic hazard. The substantially lower intraperitoneal than oral LD_50_ values and the haemolytic findings reported after oral administration of 4 g/kg/day for 90 days should be considered alongside the pharmacokinetic evidence [2]. In rats, the absolute oral bioavailability was approximately 0.14% for α-hederin and 0.118–0.250% for hederacoside C, depending on whether the isolated compound or an ivy extract was administered [8,50]. Exploratory human investigations detected no or only trace concentrations of α-hederin and did not provide a validated concentration–time profile [2]. These findings indicate that the membrane toxicity observed after very high oral exposure, direct cellular exposure, or administration that bypasses gastrointestinal absorption cannot be extrapolated directly to labelled oral treatment. The very limited oral systemic availability reduces the clinical plausibility of systemic membrane-mediated haemolysis during labelled use; however, the absence of therapeutic dose human pharmacokinetic, haematological, and exposure–response data prevents the calculation of a quantitative safety margin for this endpoint [2,8,50,81,82].

Most clinical courses last approximately one week, while dedicated chronic exposure studies are limited. The available repeated-dose evidence does not constitute a complete long-term toxicology programme for every monograph extract. Studies of isolated α-hederin administered parenterally represent a different material and route from an oral finished extract [2,35,83].

### 8.2. Genotoxicity and Carcinogenicity

Regulatory assessment identified no complete extract-specific genotoxicity package for all recognised preparations. Elias et al. reported antimutagenic testing of isolated saponins. Amara-Mokrane et al., Mba Gachou et al., and Villani et al. reported reduced induced DNA damage or micronucleus formation in cellular assays involving α-hederin. These experiments assessed protective or antimutagenic activity rather than a standard complete test battery for a finished extract [2,68,69,70,71].

In the cited reverse-mutation study, α-, β-, and δ-hederin were tested at up to 400 μg per plate in *Salmonella* strains TA97, TA98, TA100, and TA102 with and without metabolic activation and were neither toxic nor mutagenic under the reported conditions. α-Hederin at 0.013–13 nmol/mL also reduced doxorubicin-induced micronuclei in cultured human lymphocytes without a reported intrinsic clastogenic, aneugenic, or cytotoxic effect. These studies do not constitute a complete contemporary genotoxicity battery for each medicinal extract [2,68,69].

On this basis, the existing data do not provide a contemporary extract-specific genotoxicity dataset. A standardised battery should be generated under Good Laboratory Practice for each ready-to-use commercial native extract, fully characterised by botanical source, extraction solvent, drug-to-extract ratio, marker content, and chromatographic fingerprint, rather than for isolated constituents. Consistent with the Option 1 framework of ICH S2(R1), the battery should comprise a bacterial reverse-mutation (Ames) test conducted with and without metabolic activation, an in vitro mammalian assay for chromosomal damage using either the metaphase chromosome aberration or in vitro micronucleus test, and an in vivo rodent erythrocyte micronucleus test with confirmation of systemic or target tissue exposure [84,85,86]. Testing the complete extract rather than α-hederin alone is essential, since the co-extracted saponin, phenolic, and polyacetylene fractions vary among preparations and collectively constitute the active material administered to patients [1,2,3,4,5,29,30,31,85].

Dedicated carcinogenicity studies of the monograph preparations were not identified. The short intended duration of medicinal use is relevant to regulatory study requirements, but the absence of a study is recorded separately from a negative carcinogenicity result [2].

### 8.3. Reproductive and Developmental Toxicity

Daston et al. administered α-hederin subcutaneously to rats and examined maternal zinc status and developmental outcomes. Duffy et al. studied repeated subcutaneous α-hederin and zinc metabolism during development. Both experiments bypassed gastrointestinal absorption and used isolated compound exposure [35,83].

Single subcutaneous α-hederin doses of 3–300 μmol/kg were administered to pregnant rats on gestation day 8 or 11. Doses of 30 μmol/kg and above increased resorptions, while 300 μmol/kg reduced foetal weight and increased abnormal findings. Repeated subcutaneous exposure at 20–30 μmol/kg on gestation days 6–15 produced related developmental changes together with hepatic metallothionein induction and altered zinc distribution. These constituent-level parenteral studies identify a developmental hazard at systemic exposure; they do not supply a no-observed-adverse-effect level for a labelled oral extract [2,35,83].

The EMA assessment did not identify an adequate reproductive toxicity package for the authorised extracts. Clinical pregnancy data were later supplemented by a retrospective cohort, described in Section 10.2. The EU monograph does not recommend use during pregnancy or lactation [1,2,87].

### 8.4. Safety Pharmacology and Interaction-Related Toxicology

Dedicated guideline safety pharmacology studies covering cardiovascular, central nervous system, and respiratory function were limited in the regulatory dataset. A case report described dystonia in a three-year-old child after an ivy-containing cough preparation. Clinical trials and large observational studies supply additional short-term safety observations but were not designed as formal safety pharmacology studies [2,10,88].

Microsomal CYP inhibition and rodent CYP suppression constitute nonclinical interaction findings. The clinical significance has not been measured in a human interaction study [65,77,80].

Two additional systemic mechanisms have been identified in rodent studies of isolated α-hederin. First, repeated systemic exposure reduced total hepatic cytochrome P450 content and suppressed selected CYP1A1/2 and CYP2E1 measures. This effect could theoretically modify the metabolic clearance or bioactivation of concomitant xenobiotics, although its direction and clinical importance would depend on the affected substrate and the systemic α-hederin concentration [65,76,77]. Second, maternal hepatic metallothionein induction with altered zinc distribution constitutes the proposed mechanism of the developmental findings, as described in Section 8.3 [35,83]. Both mechanisms are supported under direct systemic exposure; they have not been demonstrated after labelled oral use, considering the parenteral route employed in the underlying studies, the very low oral bioavailability of α-hederin in rats, and the absence of adequate human exposure data [2,35,50,65,76,77,83]. The regulatory toxicology evidence is distributed across whole extracts, saponin mixtures, and isolated α-hederin. Oral extract studies most closely match medicinal use. Subcutaneous α-hederin studies produce systemic exposure without gastrointestinal absorption, while in vitro membrane, genotoxicity, or CYP studies expose cells or enzymes directly. Each route and material supplies a different component of the safety dataset [2,35,80,83].

Clinical safety studies add short-duration oral exposure in adults and children. Most participants were treated for approximately seven days, and many observational reports relied on spontaneous or physician-recorded adverse events. They provide greater population size than the animal studies but do not replace reproductive, genotoxicity, carcinogenicity, or long-term toxicology endpoints [2,10,13,14,38].

The principal toxicological findings for ivy extracts and α-hederin are summarised by endpoint in Table 9.

Evidence synthesis. The available animal studies indicate relatively low acute oral toxicity for the tested ivy extracts. The membrane-disrupting and haemolytic activity of triterpenoid saponins is concentration- and route-dependent, with relevant findings reported after very high oral dosing, direct cellular exposure, or administration bypassing gastrointestinal absorption. Selected hederins produced negative results in the reported bacterial reverse-mutation assays, while isolated α-hederin produced developmental toxicity in rats after subcutaneous administration through a maternally mediated mechanism involving metallothionein induction and altered zinc distribution [2,35,68,81,82,83]. These observations establish specific hazards within their respective experimental systems but do not provide a complete safety assessment of labelled oral extracts. Extract-specific genotoxicity, reproductive and developmental toxicity, duration-appropriate repeated-dose toxicity, and carcinogenicity remain insufficiently characterised under contemporary standards, and the clinical relevance of the CYP-inhibition findings has not been determined [2,65,76,77,80,84,85,86]. Favourable tolerability during short clinical treatment courses cannot substitute for these missing toxicological endpoints [2,10,13,14,38]. The principal limitation for risk assessment is the mismatch between the materials and routes used in many hazard studies—isolated constituents, direct cellular exposure, or parenteral administration—and the chemically complex extracts administered orally in clinical practice. Together with the absence of reliable therapeutic dose human exposure data, this mismatch prevents calculation of quantitative safety margins for the identified hazards [2,8,35,50,80,81,82,83,84,85,86].

## 9. Clinical Efficacy

Clinical studies of ivy mono-preparations include randomised placebo-controlled trials, randomised active comparator trials, prospective observational studies, postmarketing cohorts, retrospective database analyses, and paediatric asthma studies. Treatment periods were commonly seven days for acute cough or acute bronchitis. Outcomes included cough frequency or cough severity scales, Bronchitis Severity Score, sputum and auscultatory findings, global clinical assessment, lung function variables, antibiotic prescribing, and adverse events [10,11,12,13,40,41].

The Bronchitis Severity Score combines cough, sputum, rales or rhonchi, chest pain during coughing, and dyspnoea. It has been used as the primary or major outcome in several adult trials. Cough-specific scales and daily diaries were used in other studies. The two placebo-controlled EA 575 trials were later analysed together using individual participant data [12,13,17,89].

Studies of thyme/ivy, thyme/primrose, ivy/Coptis, and other combinations were not included as direct proof of ivy mono-preparation efficacy. The 2025 three-arm trial is included as a direct comparison between EA 575 and two herbal combinations [18].

### 9.1. Adult Clinical Studies

Schaefer et al. randomised 181 adults with acute cough in a multicentre double-blind trial. Participants received a liquid containing EA 575 or matched placebo for seven days. The prespecified cough-related outcome improved more in the EA 575 group, and the report described similar short-term tolerability between groups [12].

Cough severity was recorded on a visual analogue scale across the seven-day treatment period, with area under the curve from 0 to 168 h as the primary efficacy variable. Bronchitis Severity Score and a verbal category scale were secondary measures. The active group separated from placebo within 48 h, and the reported advantage remained at subsequent visits and at the post-treatment assessment. Adverse events occurred in 21 of 181 participants: 9 received ivy and 12 received placebo. All were non-serious, mild, or moderate and judged unrelated to study medication [12].

A second multicentre double-blind study randomised 209 adults with acute bronchitis to two EA 575 dosing schedules or placebo. Both active schedules produced larger Bronchitis Severity Score reductions than placebo. The comparison between the two active schedules supported similar efficacy under the tested dosing schemes [13].

Völp et al. obtained individual participant data from the two placebo-controlled trials. The pooled dataset contained 228 EA 575 recipients and 162 placebo recipients. At day 7, the modelled mean reduction in Bronchitis Severity Score was 8.6 points with EA 575 and 6.2 points with placebo. Cough-free status was reported in 18.1% and 9.3% of participants, respectively [17].

Cwientzek et al. enrolled 590 patients with acute bronchitis in a randomised double-blind non-inferiority comparison of two ivy extracts. Both groups improved by approximately five Bronchitis Severity Score points over seven days, and the protocol-defined non-inferiority result was met. Adverse events were reported in 2.7% of participants [11].

The Cwientzek population ranged from 2 to 86 years and received one of two defined ivy preparations for seven days. Mean baseline Bronchitis Severity Scores of approximately 6.2–6.3 fell by 4.7–4.9 points to about 1.4–1.6. Cough, sputum, rales or rhonchi, chest pain during coughing, and dyspnoea improved in both groups. Sixteen participants in each group, 2.7%, experienced an adverse event; all events were non-serious. The design demonstrates non-inferiority between the tested preparations under the study margin and does not include a no-treatment arm [11].

Kruttschnitt et al. compared an ivy cough syrup with acetylcysteine in a non-interventional multicentre study of adults and children with acute bronchitis. Clinical symptom measures improved in both groups, and tolerability was reported as similar. Treatment allocation was based on routine practice rather than random assignment [40].

Kardos et al. randomised adults with acute bronchitis to EA 575 drops, an ivy/thyme combination, or a thyme/primrose combination in an open-label multicentre trial. The modified full analysis set included 325 patients. EA 575 met the non-inferiority criteria versus both combinations. Superiority was reported versus ivy/thyme but not versus thyme/primrose. All reported adverse events were non-serious [18].

Fazio et al. conducted a prospective open postmarketing study in 9657 patients, including adults and children, with acute or chronic inflammatory bronchial disease. After seven days, 95% were classified as improved or healed in the report. The study also recorded co-medication and safety events. Stauss-Grabo et al. followed 330 patients using film-coated ivy tablets for cough associated with colds and reported symptom improvement and tolerability outcomes [10,38].

The Fazio cohort was conducted in 11 Latin American countries over 23 months and included patients from infancy to 98 years; a total of 53.7% of patients were children. Improvement or absence of the recorded clinical symptoms was reported in 95.1% of patients after treatment. Cough and expectoration improved or resolved in approximately 93%, and dyspnoea and chest pain in approximately 91%. Adverse events were recorded in 2.1% (95% CI 1.8–2.4%), 0.5% discontinued, and gastrointestinal complaints predominated. Concomitant antibiotics and other treatments were common, and the uncontrolled design does not separate treatment effect from recovery or co-medication [10].

The principal adult clinical studies of ivy mono-preparations are summarised in Table 10.

### 9.2. Paediatric Clinical Studies

The paediatric subgroup in the Fazio postmarketing study comprised 5181 patients. Children received the ivy syrup within the routine practice protocol, and respiratory findings and adverse events were recorded over seven days. The report did not include an untreated or placebo paediatric group [10].

The older controlled paediatric programme included several small studies with physiologic outcomes. In 24 children aged 4–12 years with bronchial asthma, a randomised double-blind placebo-controlled crossover study used ivy drops twice daily for three days. Airway resistance decreased by 0.14 kPa/L/s, or 23.6%, relative to placebo (*p* = 0.0361), and intrathoracic gas volume decreased by 0.16 L, or 10.1% (*p* = 0.0007); other spirometric measures were secondary. A separate 26-child crossover comparison of drops and suppositories reported improvement with both formulations but did not include placebo [42].

An open controlled study compared ivy syrup in 53 children with ambroxol in 19 children aged 7 months to 15 years. Both groups showed resolution of productive cough during 7–14 days; normalisation of selected expiratory-flow measures in children with obstruction was reported in the ivy group. Another open controlled study randomised 50 children aged 2–10 years with acute bronchitis to ivy syrup or acetylcysteine for 7–10 days. Both groups improved, while larger changes in spirometric indices were reported with ivy, including FEV1 being expressed as a percentage of the predicted amount. Allocation, masking, concomitant therapy, and reporting standards limit direct comparison with modern trials [21].

Schmidt et al. reported two open non-interventional studies using syrup and drops in 268 children with cough or bronchitis. Physician and caregiver assessments improved during treatment, and both formulations were described as well tolerated [14].

The two Schmidt cohorts contained 131 children receiving syrup and 137 receiving drops for up to 14 days. At study end, rhinitis, cough, and viscous mucus were absent or only mild in 93.0%, 94.2%, and 97.7% of participants, respectively, and the global effect was rated good or very good in 96.5%. Five adverse events were recorded, four gastrointestinal, and none serious. These percentages describe change during treated observation; neither cohort included placebo or an untreated control [14].

Lang et al. conducted a non-interventional study in 1066 schoolchildren aged 6–12 years with acute bronchitis. Five EA 575 dosage forms were used in routine practice. Physician findings, patient-reported symptoms, diary entries, tolerability, and compliance were assessed during a seven-day course; improvement was reported across the clinical measures [39].

Hofmann et al. reviewed older randomised trials of dry ivy extracts in children with bronchial asthma. The studies measured lung function during add-on therapy and reported changes in selected variables. The underlying trials used different preparations and older reporting methods [42].

Zeil et al. enrolled 30 children with controlled allergic asthma receiving inhaled corticosteroids in a randomised double-blind crossover study. Ivy dry extract or placebo was added for the study periods. The primary FEV1 and mid-expiratory flow comparisons did not show a clear significant difference; changes were reported for selected secondary flow measures [15].

Vogelberg et al. analysed prescription data for children and adolescents with common cold. Propensity score matching produced 10,390 patients in the EA 575 and antibiotic groups. Subsequent antibiotic prescribing was less frequent in the EA 575 group. The database did not contain randomised allocation, standardised cough scores, or microbiological confirmation [41].

The paediatric clinical evidence described above is summarised by study design in Table 11.

### 9.3. Published Reviews and Quantitative Synthesis

Holzinger and Chenot reviewed controlled clinical studies published through the earlier search period and concluded that the evidence was insufficient for firm efficacy conclusions. The updated review by Sierocinski et al. included six randomised trials, one controlled clinical trial, and four observational studies. All included studies reported favourable findings, no serious adverse effects were identified, and the reviewers rated reporting quality low and risk of bias high [16,22].

The individual participant meta-analysis by Völp et al. included the two double-blind placebo-controlled EA 575 trials and reported the numerical results summarised above. Lang et al. reviewed product-specific EA 575 clinical studies, including randomised and observational reports, and described efficacy and tolerability across acute and chronic respiratory conditions [17,21].

Barnes et al. performed a rapid review focused on viral respiratory infections and did not identify direct clinical evidence for an antiviral treatment effect. Baharara et al. reviewed human respiratory and cough studies across ivy preparations [23,24].

The principal published reviews and quantitative syntheses of clinical ivy evidence are compared in Table 12.

Evidence synthesis. The strongest clinical efficacy evidence concerns short-term treatment of adults with acute cough or acute bronchitis using the defined EA 575 preparation. Two double-blind placebo-controlled trials, subsequently combined in an individual participant data analysis, reported greater reductions in cough or Bronchitis Severity Score than placebo under the tested conditions [12,13,17]. Active comparator trials reported non-inferiority or comparable improvement for particular preparations but do not independently establish absolute efficacy or an extract-independent class effect. Observational and postmarketing studies provide directionally consistent reports of symptom improvement, although natural recovery, selection, concomitant treatment, and the absence of untreated controls limit causal attribution [10,11,18,38,40]. Paediatric evidence remains substantially less certain: no large modern placebo-controlled trial was identified for acute productive cough within the authorised age groups, while the modern asthma crossover study did not demonstrate a clear benefit in its primary lung function comparison and addressed an indication different from productive cough [14,15,39,41,42]. The controlled evidence is concentrated around one proprietary extract, and several pivotal publications disclose manufacturer sponsorship or manufacturer-affiliated authors, limiting the extent of independent clinical replication [12,13,17]. Translation is further constrained by treatment periods generally being limited to seven days, reliance on symptom-based scales, heterogeneous outcome definitions, and preparation specificity. The findings consequently should not be generalised to prolonged treatment, all ivy extracts, chronic respiratory disease, or paediatric efficacy as a class [10,11,12,13,14,15,16,17,18,38,39,40,41,42,89].

## 10. Clinical Safety and Tolerability

### 10.1. Clinical Adverse Events and Population Safety

Clinical trials and observational studies most often reported gastrointestinal adverse events, including nausea, vomiting, diarrhoea, abdominal discomfort, and gastric irritation. The EMA monograph also lists hypersensitivity reactions, including urticaria, skin rash, and dyspnoea. Frequencies cannot be calculated precisely from the monograph dataset [1,2,10,38].

In the 590-patient randomised extract comparison trial, 2.7% of participants experienced an adverse event. The placebo-controlled EA 575 studies reported similar short-term tolerability between active and placebo groups. The 2025 open-label head-to-head trial recorded a low incidence of adverse events across all three herbal treatment groups, and all reported events were non-serious [11,12,13,18].

The 9657-patient postmarketing study recorded a low adverse-event frequency during the seven-day treatment period. Observational tablet and paediatric studies also described predominantly mild events. These studies provide the largest treated population safety counts in the literature [10,14,38,39].

A retrospective paediatric tolerability survey collected records from 310 physicians for 52,478 children aged 0–12 years treated with ivy syrup. A total of 115 adverse drug reactions were documented, an incidence of 0.22%; gastrointestinal reactions accounted for 0.17% of reactions, including diarrhoea 0.10%, enteritis 0.04%, and vomiting 0.02%, while allergic skin reactions accounted for 0.04%. All were described as non-serious, mild, and transient. The large denominator is valuable for common event surveillance, while retrospective ascertainment can under-record events and cannot supply a controlled incidence estimate [21].

Within the prospective 9657-patient cohort, adverse events occurred in 2.1% overall and in 1.2% of children; a total of 46 patients, 0.5%, stopped treatment, mainly for gastrointestinal events. Tolerability was rated good or very good in 96.6% of patients. Concomitant antibiotic use was associated with a higher frequency of gastrointestinal complaints, although the observational design cannot allocate causality reliably between ivy, antibiotic exposure, infection, and formulation excipients [10].

Paediatric safety data were reported in the 5181-child subgroup of the Fazio cohort, the 268-child studies summarised by Schmidt et al., the 1066-schoolchild study, and the 30-child asthma crossover trial. The main recorded adverse event categories were gastrointestinal or hypersensitivity-related, and no recurring paediatric organ system signal was reported during the short treatment periods [10,14,15,39].

The EU monograph contraindicates use in children younger than two years due to the general risk that secretolytic medicines can aggravate respiratory symptoms. Persistent or recurrent cough in children aged two to four years requires medical diagnosis. Product-specific authorisations may contain additional age, dose, or excipient restrictions [1,2].

Nausea, vomiting, diarrhoea, abdominal discomfort, and gastric irritation are the main gastrointestinal reactions listed in clinical reports and the EU monograph. In most short-term studies, these events were mild and infrequent. Their attribution is not straightforward, since both the active constituents and the formulation may contribute [1,2,10,38].

Triterpenoid saponins may contribute to gastrointestinal intolerance through their surface-active properties, local interactions with mucosal cell membranes, and effects on gastric motility. The membrane–cholesterol interactions described in Section 8.1 provide biological plausibility for local irritation after direct gastrointestinal exposure, while experiments using isolated rat stomach strips indicate that ivy extract and selected constituents can modify gastric motor activity. However, direct saponin-induced mucosal injury has not been demonstrated in patients receiving labelled doses; this mechanism should therefore be considered plausible rather than clinically established [66,81,82].

Formulation excipients constitute a separate potential source of gastrointestinal symptoms. Some oral liquid products contain sorbitol, maltitol, or related incompletely absorbed polyols, which can produce dose-dependent osmotic diarrhoea, flatulence, and abdominal discomfort. In the retrospective survey of 52,478 children, 115 adverse drug reactions were recorded, corresponding to 0.22% of the treated population, and gastrointestinal reactions accounted for 0.17%. A subsequent review suggested that much of the reported diarrhoea was presumably related to the sorbitol content of the syrup; however, the absence of an excipient-matched comparator prevents confirmation of this attribution [2,21,90]. In the 9657-patient observational study, gastrointestinal complaints were more frequent among patients receiving concomitant antibiotics, providing an additional source of confounding data [10]. Because most clinical studies did not report the complete excipient composition or polyol exposure per administered dose, the respective contributions of the extract, formulation excipients, respiratory illness, and concomitant medicines cannot be quantified. Future tolerability studies should report the complete formulation and use an excipient-matched comparator whenever feasible. In clinical practice, the product information should be consulted for formulation-specific polyol and hereditary-fructose intolerance warnings [1,2,10,21,38,91].

### 10.2. Hypersensitivity, Overdose, and Special Populations

Morfin-Maciel et al. reported two cases of anaphylaxis after ingestion of ivy syrup. Pokladnikova et al. analysed allergy-like immediate reactions to herbal medicines in VigiBase and included ivy-related signals. The product, excipients, timing, concomitant medicines, and diagnostic testing vary across individual reports [91,92].

Hausen et al. identified falcarinol and didehydrofalcarinol as allergens or irritants in common ivy and documented contact dermatitis. Bach et al. reported a later contact allergy case. These reports concern fresh plant or skin exposure rather than the oral clinical trials of dried-leaf extract [33,34].

Polizzi et al. reported an acute dystonic reaction in a three-year-old child after arbitrary maternal administration of a preparation containing methylcodeine and hedera extract. The report establishes a temporal association with the combination product but does not establish ivy-specific causality or provide an incidence estimate [88].

The EMA monograph does not describe a characteristic therapeutic overdose syndrome from the clinical trial programme. Management of significant accidental ingestion is supportive and guided by symptoms, product composition, quantity, age, and poison centre advice [1,2].

Alkattan et al. conducted a retrospective cohort study involving 165 ivy-exposed pregnancies and 80 controls. The study did not detect a statistically clear increase in the reported maternal or neonatal outcomes. Exposure was retrospective, and the sample was not powered to exclude uncommon outcomes [87].

Safety boundaries also include accidental intake and special populations. The regulatory assessment records nausea, vomiting, diarrhoea, and agitation after overdose and describes a four-year-old child who developed aggression and diarrhoea after an intake corresponding to approximately 1.8 g of herbal substance. A separate case report described an acute dystonic reaction after an ivy-containing cough product. Two anaphylaxis cases and allergy-like pharmacovigilance reports establish that immediate hypersensitivity, while uncommon, is clinically relevant. No prospective safety dataset is adequate to support pregnancy, lactation, severe organ impairment, or prolonged continuous use [2,83,91,92].

The EMA monograph states that safety during pregnancy and lactation has not been established and does not recommend use. This position also reflects the incomplete reproductive toxicity dataset described in Section 8.3 [1,2].

Evidence for several other special population scenarios is absent rather than reassuring. No study has evaluated ivy leaf extracts during chronic use in patients with hepatic or renal impairment, and the rodent cytochrome P450 findings (Section 7.2 and Section 8.4) have not been examined in such patients, so a theoretical concern remains unquantified. Likewise, no dedicated data address older adults receiving polypharmacy, in whom additive or opposing effects are plausible. From a pharmacodynamic standpoint, an expectorant that mobilises bronchial secretion is mechanistically opposed by centrally acting antitussives such as codeine or dextromethorphan, because suppressing the cough reflex may impair clearance of the mobilised secretions; the EU monograph therefore advises against combining ivy preparations with these antitussives without medical supervision, and concurrent use with other mucolytics has not been formally studied [1,2]. These situations represent gaps in the evidence base rather than demonstrated harms and should be presented as such when prescribing for elderly or organ-impaired patients.

### 10.3. Exposure Limits and Clinical Warnings

The EU monograph advises consultation with a physician or pharmacist if symptoms persist longer than one week during use. Dyspnoea, fever, or purulent sputum requires medical assessment. Product-specific leaflets provide age limits, extract dose, dosage-form instructions, and excipient warnings [1,2].

Randomised trials contribute comparator-based adverse event counts for several hundred adults. Observational and postmarketing studies contribute larger treated populations, including more than 5000 children in the Fazio cohort and more than 1000 schoolchildren in the Lang study. Case reports and VigiBase analyses contribute information about uncommon hypersensitivity or neurologic events [10,11,12,13,39,83,91,92].

Most efficacy and safety studies evaluated one week of treatment, with some follow-up to two weeks. The literature contains less information on continuous use over months, repeated courses in the same patient, organ impairment, and complex polypharmacy [2,10,13,18].

The clinical safety evidence and corresponding regulatory precautions are summarised in Table 13.

### 10.4. Comparison with Other Respiratory Phytotherapeutics

Ivy occupies a distinct position among European respiratory phytotherapeutics. The EMA/HMPC recognises specified ivy leaf preparations under well-established use as expectorants in productive cough. By comparison, the thyme herb and primula root monographs rely on traditional use, and the pelargonium root monograph is likewise based on traditional use and concerns symptomatic treatment of the common cold rather than an identical productive cough indication; ivy leaf is thus the only agent among these four with well-established-use status. At the time of the corresponding HMPC assessments, the evidence for thyme monotherapy was insufficient for firm clinical conclusions, and no adequate clinical studies of primula root monotherapy were available. Trials of pelargonium preparations in acute respiratory infections suggested possible benefit, but extract specificity and methodological limitations restricted the conclusions that could be drawn [1,2,93,94,95,96].

Direct comparative clinical evidence remains limited. In the 2025 open-label study, EA 575 was non-inferior to ivy/thyme and thyme/primula combinations for the change in Bronchitis Severity Score after seven days. Statistical superiority was reported against the ivy/thyme combination but not against the thyme/primula combination. The open-label design, short follow-up, and use of specific proprietary preparations prevent extrapolation to all ivy, thyme, or primula products and do not establish an overall therapeutic ranking [18].

Gastrointestinal complaints and hypersensitivity reactions occur across several respiratory herbal products, although the frequency, definitions, and ascertainment methods vary. Thyme preparations may cause gastric complaints or allergic reactions; safety information for primula root monotherapy remains comparatively sparse; and pelargonium products carry a separate indication and product-specific precautions. No harmonised comparative dataset covering repeated-dose toxicity, genotoxicity, reproductive toxicity, or pharmacological interactions was identified across these botanicals, so comparative toxicological superiority cannot be claimed for any preparation. Differences in extraction procedure, chemical composition, dose, age restrictions, indication, and outcome definition further preclude a quantitative cross-product benefit–risk ranking. Appropriately blinded head-to-head trials using chemically characterised preparations and common efficacy and adverse event definitions remain necessary [18,93,94,95,96].

Evidence synthesis. Across controlled studies, large observational and postmarketing cohorts, and regulatory assessment, short-term oral tolerability is generally favourable. Gastrointestinal reactions were predominantly mild, while hypersensitivity was reported infrequently but included potentially serious immediate reactions [1,2,10,13,14,38,90,91,92]. Incidence estimates are not directly comparable because adverse event ascertainment ranged from structured trial recording to physician reporting and spontaneous surveillance, with the largest population estimates derived mainly from uncontrolled studies. Causal attribution of gastrointestinal events also remains uncertain: triterpenoid saponins, polyol excipients, respiratory infection, antibiotics, and other concomitant medicines may all contribute [1,2,10,21,38,66,81,82,90]. Evidence remains inadequate for prolonged or repeated use; pregnancy evidence is limited to retrospective observation; and no dedicated studies address lactation, moderate, or severe hepatic or renal impairment, or older adults receiving polypharmacy [1,2,65,80,87]. Potential interactions involving cytochrome P450 inhibition, centrally acting antitussives, or concomitant mucolytics have not been confirmed in controlled clinical coadministration studies [1,2,40,65,80]. The heterogeneous evidence available for ivy, thyme, primula, and pelargonium also does not support a quantitative comparative safety ranking [18,93,94,95,96]. The principal limitation of the clinical safety evidence is its predominantly one-week exposure horizon and the reliance on uncontrolled designs for population-level incidence estimates. The findings support generally favourable tolerability during labelled short-course use but do not establish safety during chronic exposure or in inadequately studied special populations [1,2,10,13,14,38].

## 11. Regulatory Status

The EMA Committee on Herbal Medicinal Products finalised Revision 2 of the assessment report and European Union herbal monograph in 2018. Five ivy leaf preparation categories are listed for well-established medicinal use as expectorants in productive cough. Posology is specified by preparation and age group. The monograph contains contraindications, adverse reaction wording, duration advice, and pregnancy/lactation statements [1,2].

Regulatory recognition applies to five preparation categories rather than to every ivy-labelled product. Adult and adolescent daily extract doses vary by preparation, as do the authorised paediatric doses. The monograph contraindicates use below two years, requires medical evaluation for persistent or recurrent cough at two to four years, advises review when symptoms persist beyond one week, and identifies dyspnoea, fever, or purulent sputum as reasons for clinical assessment. It also recommends caution in gastritis or gastric ulcer and advises against combining an expectorant with codeine or dextromethorphan without medical guidance [1,2].

EMA issued a call for scientific data in 2025 for a periodic review of the monograph. The consultation closed on 31 May 2025. No newer final EU monograph was publicly available at the search cut-off [97].

The ESCOP monograph describes ivy leaf use in respiratory conditions with productive cough and provides dosage, contraindication, safety, clinical, and pharmacological information available at the time of publication [36].

The German Commission E monograph recognised ivy leaf for catarrhal respiratory complaints. The Commission E and ESCOP documents preceded the harmonised European Union monograph and form part of the historical regulatory development of ivy phytotherapy [36,37].

The European Pharmacopoeia monograph defines *Hederae folium* and requires no less than 3.0% hederacoside C in the dried drug. Finished medicinal products remain subject to national or regional authorisation, manufacturing, stability, labelling, and pharmacovigilance requirements [2,3].

The EU monograph provides a common scientific basis for the specified preparations, while marketed products differ in exact extract, formulation, concentration, excipients, and authorised age range. Clinical evidence reported for EA 575, another defined extract, or a combination preparation remains linked to that intervention [1,11,12,18].

National authorisations may include syrups, oral drops, film-coated tablets, and effervescent or other solid dosage forms. The package information identifies the extract quantity per dose and the approved paediatric or adult schedule [1,2,38,39].

Ivy products are marketed in multiple regions under medicinal product, traditional-medicine, or supplement frameworks. Evidence requirements, permissible claims, age limits, and labelling therefore differ by jurisdiction. The present review uses the EU monograph as the main regulatory reference due to its public preparation-specific assessment [1,2].

The 2025 periodic review process provides a route for evaluating the recent animal cough study, dendritic cell study, comparative acute bronchitis trial, paediatric prescription analysis, pregnancy cohort, and other evidence published after Revision 2. Human pharmacokinetics and extract comparability data remain absent from the public literature [7,18,41,56,82,97].

The principal regulatory and pharmacopoeial documents relevant to ivy leaf medicines are summarised in Table 14.

Evidence synthesis. At the review cut-off, the European regulatory status of ivy leaf medicines was well defined. The EU herbal monograph recognised five specified preparation categories—three dry extracts, one liquid extract, and one soft extract—under well-established use as expectorants in productive cough and established preparation- and age-related posology, contraindications, and warnings. Ph. Eur. Issue 12.2 provided the corresponding official quality standard for *Hederae* folium [1,2,3]. These constitute established regulatory and pharmacopoeial requirements but remain preparation-specific; they do not demonstrate therapeutic equivalence or interchangeability among extracts, finished products, or products authorised in different jurisdictions. In particular, no defined regulatory bridging criteria connect preparations differing in drug–extract ratio, extraction solvent, and chemical composition to a common body of efficacy and safety evidence [1,2,3,29,30,31]. The principal open regulatory question at the search cut-off was the outcome of the periodic review initiated by the 2025 EMA call for scientific data; no later final EU herbal monograph was identified by June 2026 [97]. The practical limitation is therefore the discrepancy between preparation-specific regulatory recognition and generic references to “ivy” in clinical discussion. Prescribing, evidence transfer, and any consideration of substitution should be based on the exact authorised preparation rather than an assumed class-wide equivalence [1,2].

## 12. Knowledge Gaps and Future Perspectives

The evidence inventory identifies a large mechanistic literature and a small pharmacokinetic literature. Human studies are needed to measure hederacoside C, α-hederin, hederagenin, and conjugated metabolites after labelled doses. Sampling after single and repeated administration should be paired with validated stability procedures and documentation of the clinical batch [8,9,50,79].

Nonclinical respiratory studies should report complete extract composition, dose–response data, randomisation, blinding, sample-size justification, and measured plasma or tissue exposure. Independent replication of β2 receptor, A2B receptor, immune cell, and 2025 cough model results would expand the number of research groups contributing to these mechanisms [6,7,55,56].

Clinical research priorities include an independent placebo-controlled adult trial, placebo-controlled paediatric studies within authorised age groups, objective cough frequency measurement, responder thresholds, longer follow-up, and reporting by extract and formulation. Head-to-head studies require full chemical description of both interventions [11,16,17,18].

Safety research priorities include oral extract-specific developmental and reproductive toxicity, an updated, ICH S2(R1)-compliant genotoxicity battery performed under Good Laboratory Practice on ready-to-use commercial extracts (bacterial reverse mutation, an in vitro chromosomal damage assay, and an in vivo micronucleus test), duration-appropriate repeated-dose testing, and clinical assessment of the CYP inhibition signal after human exposure is known. A prospective pregnancy registry would complement the existing retrospective cohort [2,80,84,85,86,87].

Quality research should compare EMA preparation categories using hederacoside C, α-hederin, broader saponin and phenolic fingerprints, dissolution, and stability. These data could support defined evidence-bridging criteria between products [1,29,30,31].

The principal evidence gaps and corresponding proposed study designs are summarised in Table 15.

## 13. Conclusions

The published evidence on *H. helix* covers a defined medicinal leaf, multiple hydroethanolic extract categories, a chemically diverse constituent profile, respiratory and secondary pharmacodynamic studies, animal pharmacokinetics, toxicology, adult and paediatric clinical studies, postmarketing safety, and European regulation. Hederacoside C is the main quality marker, and α-hederin is the constituent most frequently studied in β2-adrenergic receptor models. Recent studies have added A2B receptor, dendritic cell, acute lung inflammation, animal cough, and tracheobronchial secretion data.

Rat studies have quantified hederacoside C, hederacoside D, and α-hederin, while no therapeutic dose human pharmacokinetic study was identified. The clinical evidence includes two placebo-controlled adult EA 575 trials, randomised comparisons between herbal preparations, large observational cohorts, paediatric studies, systematic reviews, and a two-trial individual participant meta analysis.

Short-term oral treatment was generally well tolerated in the reported studies. Gastrointestinal symptoms and hypersensitivity reactions are the principal recognised adverse reactions. The EU monograph authorises defined preparations as expectorants in productive cough, contraindicates use below two years, and does not recommend use during pregnancy or lactation. Human pharmacokinetics, independent clinical replication, paediatric placebo-controlled evidence, extract comparability, and modern toxicology remain the main areas for further work.

## Figures and Tables

**Table 1:** Scope of major previous *Hedera helix* reviews and the present evidence overview.

Publication	Main Scope	Evidence Domains Emphasised	Coverage Added in the Present Review
Lutsenko et al., 2010 [19]	Medicinal plant review	Botany, chemistry, traditional use, pharmacology	Post-2010 mechanisms, PK, clinical trials, safety, and current regulation
Sierocinski et al., 2021; Holzinger and Chenot, 2011 [16,22]	Systematic clinical reviews	Controlled and observational respiratory studies	Study-level nonclinical, PK, toxicology, quality, and 2025 evidence
Lang et al., 2015 [21]	EA 575 clinical review	Product-specific efficacy and tolerability	Other extracts, independent reviews, PK, and nonclinical studies
Al-Snafi, 2018 [20]	Broad pharmacological review	Multiple experimental activities	Respiratory study detail, modern mechanisms, PK, and clinical tables
Barnes et al., 2020 [23]	Rapid viral respiratory review	Human viral respiratory evidence	Complete respiratory and medicinal product evidence base
Baharara et al., 2021 [24]	Human respiratory effects	Cough and respiratory clinical studies	Detailed quality, nonclinical, PK, toxicology, and regulatory chapters
Shokry et al., 2022 [25]	*H. helix* leaf-extract review	Phytochemical content, biological activities, and therapeutic applications	Preparation-linked pharmacokinetics, toxicology, respiratory clinical evidence, safety, and current regulation
Osama et al., 2023 [26]	Genus-wide comprehensive review	Phytoconstituents, diverse pharmacological activities, and medicinal properties across *Hedera*	Respiratory medicine focus; preparation-specific quality-to-clinic assessment; pharmacokinetics, toxicology, adult and paediatric efficacy, clinical safety, and regulation; evidence updated through June 2026

PK, Pharmacokinetics.

**Table 2:** Taxonomic classification and medicinal material of *Hedera helix* L.

Category	Classification or Description
Kingdom	Plantae
Order	Apiales
Family	Araliaceae
Genus	Hedera
Species	*Hedera helix* L.
Herbal drug	Whole or cut dried leaves collected in spring
Pharmacopoeial assay	Not less than 3.0% hederacoside C in the dried drug

Sources: Plants of the World Online, European Pharmacopoeia, and EMA assessment [2,3,27].

**Table 4:** Ivy leaf preparations listed in the EU monograph and reported medicinal use.

Preparation	DER	Extraction Solvent	Dosage-Form Context	Monograph Indication
Dry extract A	4–8:1	Ethanol 24–30% *m*/*m*	Oral liquid or solid products	Expectorant in productive cough
Dry extract B	6–7:1	Ethanol 40% *m*/*m*	Oral liquid or solid products	Expectorant in productive cough
Dry extract C	3–6:1	Ethanol 60% *m*/*m*	Oral liquid or solid products	Expectorant in productive cough
Liquid extract	1:1	Ethanol 70% *V*/*V*	Oral liquid	Expectorant in productive cough
Soft extract	2.2–2.9:1	Ethanol 50% *V*/*V* with propylene glycol (98:2)	Oral liquid or solid products	Expectorant in productive cough

Data source: EMA monograph and assessment report. DER, Drug–extract ratio [1,2].

**Table 5:** Variables reported in studies of ivy leaf quality and standardisation.

Variable	Reported Assessment	Observed or Expected Effect	Reference
Species and plant part	Botanical and microscopic identity	Confirms *H. helix* leaf and excludes other plant material	[2,3]
Geographic origin	Multi-region phytochemical profiling	Variation in phenolics, saponins, amino acids, and antioxidant measures	[28]
Extraction solvent and DER	Monograph preparation definition	Changes the extracted matrix and administered extract amount	[1,2]
Hederacoside C	Pharmacopoeial assay and HPLC methods	Primary identity/Quantity marker	[3,4]
α-Hederin	HPLC or LC–MS/MS measurement	Documents an experimentally active saponin and process-related conversion	[5,30]
Multi-analyte fingerprint	Saponin, flavonoid, and phenolic profiling	Compares raw material, extract, batches, and products	[5,29]
Dosage form/Excipients	Product specification and labelling	Determines extract dose and formulation-specific tolerability	[1,2]

DER, Drug–extract ratio; HPLC, high-performance liquid chromatography; LC–MS/MS, liquid chromatography–tandem mass spectrometry.

**Table 8:** Pharmacokinetic and interaction studies of ivy constituents and extracts.

Species/System	Test Material	Route/Design	Measured Result	Reference
Rat	Hederacoside C/AG NPP709	Preclinical dosing with serial plasma sampling	IV clearance 1.46–2.08 mL/min/kg; Vss 138–222 mL/kg; oral bioavailability 0.118–0.250%; nonlinear exposure at the highest extract dose	[8]
Rat	α-Hederin	Oral and intravenous	Oral bioavailability ~0.14%; oral t1/2~2.67 h	[50]
Rat	Hederacosides C/D and α-hederin	Multi-analyte plasma PK	Different concentration–time profiles for three saponins	[9]
Rat plasma assay	Hederacoside C	Validated UHPLC–MS/MS method	Sensitive quantitative bioanalysis	[79]
Human liver microsomes	Ivy extracts	Concentration- and time-dependent inhibition experiments	CYP2C8/2C19/2D6 inhibition signals	[80]
Humans	Authorised/Proprietary preparations	Efficacy and safety studies without concentration sampling	Clinical outcomes reported	[2,16]
1 + 4 + 16 volunteers	Dry extract; α-hederin assay	Single and repeated oral administration	Mostly undetectable or near-limit α-hederin; 1.39–1.51 nmol/L in 2/16 volunteers	[2]

CYP, Cytochrome P450; PK, pharmacokinetics; t1/2, elimination half-life; UHPLC–MS/MS, ultra-high-performance liquid chromatography–tandem mass spectrometry; Vss, apparent volume of distribution at steady state.

**Table 9:** Summary of toxicological evidence for ivy extracts and α-hederin.

Endpoint	Reported Finding	Reference
Acute toxicity	Low acute oral toxicity relative to therapeutic dosing; high-dose gastrointestinal effects	[2]
Repeated dose	Haemolytic finding at a very high dose in a 90-day study; interaction of triterpenoid saponins with erythrocyte membrane cholesterol provides a plausible mechanism	[2,81,82]
Genotoxicity	No intrinsic positive signal in cited assays; protection against induced damage reported	[68,69,70,71]
Development	Altered zinc-related maternal/developmental measures	[35,83]
Carcinogenicity	No dedicated study available	[2]
Safety pharmacology	No recurring organ system signal reported during short clinical use	[2,10]
CYP interaction	CYP inhibition or suppression reported	[65,80]

CYP, Cytochrome P450.

**Table 10:** Main adult clinical studies of ivy mono-preparations.

Design/Sample	Intervention and Comparator	Duration/Outcome	Efficacy Result	Safety Result	Reference
Randomised double-blind placebo-controlled; *n* = 181	EA 575 liquid versus placebo	7 days; acute-cough score	Greater improvement with EA 575	Comparable short-term pattern	[12]
Randomised double-blind placebo-controlled; *n* = 209	Two EA 575 schedules versus placebo	7 days; BSS	Both schedules improved BSS more than placebo	Similar between schedules and placebo	[13]
Randomised double-blind non-inferiority; *n* = 590	Ivy extract versus another ivy extract	7 days; BSS	Both improved by ~5 BSS points; non-inferiority met	AE in 2.7% overall	[11]
Randomised open-label 3-arm; mFAS *n* = 325	EA 575 versus ivy/thyme and thyme/primrose	7 days primary BSS comparison; follow-up to day 14	Non-inferior to both; superior to ivy/thyme only	Low AE incidence; all non-serious	[18]
Randomised double-blind double-dummy; *n* = 99	Ivy drops versus ambroxol 30 mg tablets	4 weeks; spirometry, findings, diaries	Both groups improved; vital capacity +9.5% with ivy and +1% with ambroxol	Tolerability rated good to excellent	[21]
Non-interventional active comparison; adults/children	Ivy syrup versus acetylcysteine	Acute bronchitis; symptom assessment	Improvement reported in both groups	Comparable tolerability	[40]
Observational; *n* = 330	Ivy film-coated tablets; no control	Cold-associated cough; symptom/tolerability assessment	Symptoms improved during treatment	Generally well tolerated	[38]
Prospective open postmarketing; *n* = 9657 mixed ages	Ivy syrup; no control	7 days; physician-assessed bronchial symptoms	95% classified as improved or healed	Low reported AE frequency; gastrointestinal events predominated	[10]

AE, Adverse event; BSS, Bronchitis Severity Score; mFAS, modified full analysis set. Row shading indicates study design: blue, randomised controlled trials; yellow, prospective observational and non-interventional studies; green, postmarketing and surveillance studies.

**Table 11:** Paediatric clinical evidence for ivy leaf preparations.

Design/Sample	Population/Intervention	Outcomes	Reported Result	Safety Observation	Reference
Randomised double-blind crossover; *n* = 30	Controlled allergic asthma; ivy add-on versus placebo	FEV1 and expiratory flow variables	No clear primary benefit; selected secondary changes	Add-on treatment was tolerated	[15]
Randomised double-blind crossover; *n* = 24	Children 4–12 years with asthma; ivy drops versus placebo	Airway resistance, ITGV, spirometry	Raw −23.6% (*p* = 0.0361); ITGV −10.1% (*p* = 0.0007) versus placebo	Short exposure; no major event reported	[42]
Open controlled; *n* = 50	Children 2–10 years; ivy versus acetylcysteine	Symptoms and spirometry over 7–10 days	Both improved; larger reported spirometric changes with ivy	Reported ratings favoured ivy; limited reporting	[21]
Open controlled; *n* = 72	Children 7 months–15 years; ivy *n* = 53 versus ambroxol *n* = 19	Symptoms and spirometry over 7–14 days	Productive cough improved in both; selected flow normalisation reported with ivy	No major safety signal described	[21]
Two open non-interventional studies; *n* = 268	Children with cough/bronchitis; syrup or drops	Cough and clinical findings	Improvement reported with both formulations	Both described as well tolerated	[14]
Non-interventional; *n* = 1066	Schoolchildren 6–12 years; five EA 575 forms	Physician findings, symptoms, diary, compliance	Improvement across recorded measures	High reported tolerability/compliance	[39]
Retrospective matched database; 10,390/group	Children/adolescents with common cold; EA 575 versus antibiotic prescription	Subsequent antibiotic prescribing	Lower subsequent prescribing in EA 575 group	Database study; no standardised AE collection	[41]
Open postmarketing; *n* = 5181 children within total cohort	Acute/chronic inflammatory bronchial disease; ivy syrup	Physician-assessed symptoms; AEs	Symptoms improved over 7 days	Low reported AE rate	[10]

AE, Adverse event; FEV1, forced expiratory volume in 1 s; ITGV, intrathoracic gas volume; Raw, airway resistance. Row shading indicates study design: blue, randomised controlled trials; grey, open controlled studies without stated randomisation; yellow, prospective observational and non-interventional studies; green, postmarketing, surveillance and database studies.

**Table 12:** Reviews and quantitative syntheses of clinical ivy evidence.

Method/Scope	Included Evidence	Main Reported Conclusion	Update Supplied Here	Reference
Systematic review of acute URTI trials	Controlled ivy studies available at the time	Evidence insufficient for firm conclusion	Later placebo trials and recent studies included	[22]
Narrative review of EA 575	Randomised and non-interventional EA 575 studies	Efficacy and favourable benefit–risk described	Independent review findings and post-2015 studies added	[21]
Rapid review of viral respiratory infections	Human ivy respiratory studies relevant to viral illness	No direct clinical antiviral evidence	Maintains separation of symptom efficacy and antiviral activity	[23]
Updated systematic review	6 RCTs, 1 CCT, 4 observational studies	Low reporting quality/high RoB; benefit minimal at best and clinical importance uncertain	Adds 2022 meta-analysis and 2025 clinical studies	[16]
Human respiratory/cough review	Adult and paediatric studies	Respiratory outcomes summarised	Adds current PK, toxicology, and preparation detail	[24]
IPD meta-analysis	2 placebo-controlled EA 575 RCTs; *n* = 390	BSS −8.6 versus −6.2; cough-free 18.1% versus 9.3% at day 7	Places result beside entire adult and paediatric evidence base	[17]

BSS, Bronchitis Severity Score; CCT, controlled clinical trial; IPD, individual participant data; RCT, randomised controlled trial; RoB, risk of bias; URTI, upper respiratory tract infection.

**Table 13:** Clinical safety evidence and regulatory precautions.

Safety Topic	Observed or Stated Finding	Current Precaution	Reference
Gastrointestinal events	Nausea, vomiting, diarrhoea, abdominal discomfort	Stop or seek advice if clinically significant or persistent	[1,10,38]
Hypersensitivity	Rash, urticaria, dyspnoea, and rare anaphylaxis reports	Contraindicated in allergy to ivy/Araliaceae; urgent care for systemic reaction	[88,89]
Contact allergy	Falcarinol-related irritant or allergic dermatitis	Avoid skin exposure in sensitised individuals	[33,34]
Children <2 years	Risk from secretolytic medicines	Contraindicated	[1]
Children 2–4 years	Persistent/Recurrent cough may require diagnosis	Medical assessment before repeated treatment	[1]
Pregnancy/lactation	No clear cohort signal; overall data insufficient	Use not recommended	[2,82]
Hepatic or renal impairment	No recurring hepatic or renal signal during short-term studies	Individual clinical assessment	[2,10,11,12,13,38]
Older adults and polypharmacy	No dedicated subgroup studies	Review comorbidities, organ function, formulation excipients and concomitant medicines, particularly narrow therapeutic index treatments	[1,2,65,80]
Interactions	CYP inhibition/suppression signals; no clinical trial	Review concomitant medicines when clinically relevant	[65,80]
Duration/Red flags	Review if >1 week; assess dyspnoea, fever, purulent sputum	Seek clinical evaluation	[1]
Large paediatric retrospective survey	115 ADRs (0.22%): gastrointestinal 0.17%; allergic skin reactions 0.04%	Retrospective ascertainment; interpret as documented reporting frequency	[2,21]

ADR, adverse drug reaction; CYP, Cytochrome P450. Precautions should be checked against the authorised information for the exact product and jurisdiction.

**Table 14:** Principal regulatory and pharmacopoeial documents relevant to ivy leaf medicines.

Organisation	Document	Year/Status	Main Content	Reference
European Pharmacopoeia	*Hederae folium*, monograph 2148	12th edition, 2025	Identity and quality of dried ivy leaf; ≥3.0% hederacoside C	[3]
German Commission E	Ivy leaf monograph	Historical national monograph	Use in catarrhal respiratory conditions	[37]
ESCOP	*Hederae helicis folium* monograph	2nd edition, 2003	Respiratory use, dosage, clinical and pharmacological evidence	[36]
EMA/HMPC	Assessment report, Revision 2	Final 2018	Quality, nonclinical, clinical, safety, and regulatory assessment	[2]
EMA/HMPC	EU herbal monograph, Revision 2	Final 2018	Defined preparations, productive-cough indication, posology, warnings	[1]
EMA/HMPC	Periodic-review call for data	2025 consultation	Request for new evidence; no later final monograph at search cut-off	[97]

HMPC, Committee on Herbal Medicinal Products.

**Table 15:** Evidence gaps and proposed study designs.

Evidence Area	Current Dataset	Missing Information
Human pharmacokinetics	Rat hederacoside C/D and α-hederin studies [8,9,50]	Therapeutic dose human parent/metabolite exposure
PK–PD relationship	Cell/tissue β2 findings and animal cough/secretion models [6,7,43]	Concentration linked to human target or outcome
Adult efficacy	Two EA 575 placebo RCTs [12,13]	Independent replication and patient-noticeable responder estimate
Paediatric efficacy	Mainly observational studies; small asthma crossover [14,15,39]	Placebo-controlled age-specific benefit
Extract comparability	Marker assays and one two-extract comparison [11,30]	Criteria for transfer of clinical evidence
Reproductive/long-term safety	Parenteral α-hederin studies and limited extract data [2,35,83]	Oral extract developmental and duration-matched safety
Genotoxicity	Reverse mutation, antimutagenic, and micronucleus-related studies of isolated hederins; no complete GLP programme for chemically characterised commercial ivy extracts [2,68,69,70,71]	GLP bacterial reverse-mutation assay, in vitro mammalian chromosomal damage assay using the metaphase chromosome aberration or in vitro micronucleus test, and in vivo mammalian erythrocyte micronucleus assay using fully characterised commercial native extracts, with confirmation of systemic or target tissue exposure [84,85,86]
Drug interactions	Microsomal inhibition and rodent CYP suppression [65,80]	Clinical exposure margin and interaction magnitude
Pharmacovigilance	Trials, cohorts, case reports [10,87,88]	Product-/Batch-specific incidence and rare signals

CYP, Cytochrome P450; GLP, Good Laboratory Practice; PD, pharmacodynamics; PK, pharmacokinetics; RCT, randomised controlled trial.

## Data Availability

No new data were created or analysed in this study. Data sharing is not applicable to this article.
